# Discovery of a potent protein kinase D inhibitor: insights in the binding mode of pyrazolo[3,4-*d*]pyrimidine analogues†
†Electronic supplementary information (ESI) available. See DOI: 10.1039/c6md00675b


**DOI:** 10.1039/c6md00675b

**Published:** 2017-02-09

**Authors:** Klaas Verschueren, Mathias Cobbaut, Joachim Demaerel, Lina Saadah, Arnout R. D. Voet, Johan Van Lint, Wim M. De Borggraeve

**Affiliations:** a Department of Chemistry , Molecular Design and Synthesis , KU Leuven , Celestijnenlaan 200F , 3001 Leuven , Belgium . Email: wim.deborggraeve@kuleuven.be; b Department of Cellular and Molecular Medicine , Laboratory of Protein Phosphorylation and Proteomics , KU Leuven , Herestraat 49 box 901 , 3000 Leuven , Belgium; c Department of Chemistry , Laboratory of Biomolecular Modeling and Design , KU Leuven , Celestijnenlaan 200G , 3001 Leuven , Belgium

## Abstract

In this study, we set out to rationally optimize PKD inhibitors based on the pyrazolo[3,4-*d*]pyrimidine scaffold. The lead compound for this study was 1-NM-PP1, which was previously found by us and others to inhibit PKD. In our screening we identified one compound (3-IN-PP1) displaying a 10-fold increase in potency over 1-NM-PP1, opening new possibilities for specific protein kinase inhibitors for kinases that show sensitivity towards pyrazolo[3,4-*d*]pyrimidine derived compounds. Interestingly the observed SAR was not in complete agreement with the commonly observed binding mode where the pyrazolo[3,4-*d*]pyrimidine compounds are bound in a similar fashion as PKD's natural ligand ATP. Therefore we suggest an alternate binding mode where the compounds are flipped 180 degrees. This possible alternate binding mode for pyrazolo[3,4-*d*]pyrimidine based compounds could pave the way for a new class of specific protein kinase inhibitors for kinases sensitive towards pyrazolo[3,4-*d*]pyrmidines.

## Abstract 2

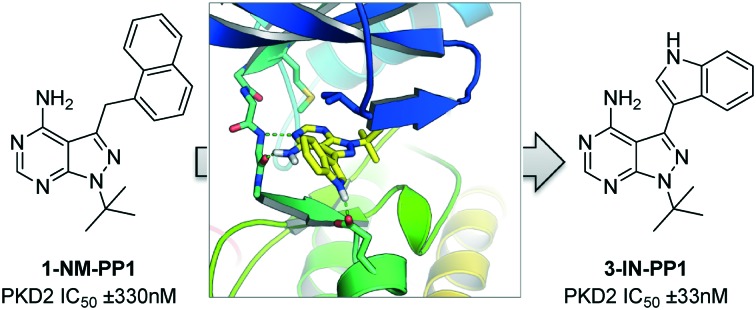
Herein we report the synthesis of pyrazolo[3,4-*d*]pyrimidine analogues of 1-NM-PP1 and the identification of 3-IN-PP1 as a new potent PKD inhibitor suggesting an alternate binding mode to PKD.

## Introduction

The Protein kinase D (PKD) family of serine/threonine kinases belongs to the broader group of the Ca^2+^/calmodulin-dependent protein kinases (CAMKs).^1^ PKDs have attracted a lot of attention due to their crucial involvement in the pathogenesis of heart hypertrophy,^2^ different cancers, tumor angiogenesis^3^ and other pathologies.^4^ The PKD family consists of 3 isoforms (PKD1/PKCμ, PKD2 and PKD3/PKCυ) with a high degree of homology especially in the catalytic domain.^5^ The activity of PKD is in many cases regulated by a diacylglycerol (DAG) induced, protein kinase C (PKC)-dependent mechanism wherein PKD is activated *via* direct phosphorylation of two conserved activation loop serine residues (Ser^738^ and Ser^742^) by PKC isoforms. Full, sustained PKD activation is achieved by subsequent auto-phosphorylation.

PKD plays a well-documented and crucial role in many important cellular processes, including cell proliferation,^6^ cell survival,^7^ gene expression,^8^ protein trafficking,^9^,^10^ cell motility^11^ and immune responses.^12^ These processes are known to play a key role in tumor biology.^4^ Interestingly, the three isoforms have differential functions in the context of various cancers. PKD1 is mainly associated with tumor suppressive functions, since it decreases cell motility and epithelial-mesenchymal transition (EMT).^13^ To this extent, PKD1 phosphorylates substrates involved in actin remodeling, such as cortactin, the cofilin phosphatase slingshot 1 L (SSH1L) and PAK4 rendering tumor cells less mobile.^14^–^18^ Furthermore PKD1 inhibits EMT *via* phosphorylation of the Snail transcription factor, resulting in decreased E-cadherin repression and hence decreased cell–cell adhesion.^19^,^20^ PKD1 also inhibits EMT *via* β-catenin phosphorylation, reducing its nuclear functions.^21^ The tumor-suppressive role of PKD1 is also reflected in its expression level in various cancers. For example in androgen-independent prostate cancer and pancreatic cancer PKD1 expression is downregulated.^22^–^24^ In breast cancer and gastric cancer PKD1 expression is down-regulated through promoter hypermethylation.^25^,^26^ In contrast, PKD2 and PKD3 exert cancer promoting properties, since they are necessary for the regulation of genes and proteins involved in metastasis and invasion. For example in pancreatic cancer cells, PKD2 regulates both the expression and secretion of matrix metalloproteinases (MMPs) 7/9.^24^ In prostate cancer cells PKD2 and 3 also increase the expression of MMP9 and the urokinase-type plasminogen activator (uPA) *via* NF-κB and HDAC1.^27^ This results in breakdown of extracellular matrix (ECM), consequently increasing cancer cell invasiveness. Furthermore, ECM breakdown results in the release of VEGF-A which is sequestered in the ECM upon secretion.^24^ In pancreatic and gastric tumor cells, PKD2 has also been shown to increase the angiogenic response to hypoxic conditions *via* its actions in both the endothelial cell as well as the cancer cell.^28^ PKD1 and 2 are both expressed in endothelial cells. However, there are several indications that PKD2 may be more important. Firstly, PKD2 is more abundantly expressed in HUVECs when compared to PKD1.^29^ Only PKD2 knockdown was able to inhibit endothelial proliferation, migration and tube formation in response to serum.^29^ Azoitei *et al.* showed that PKD2 is highly expressed in a variety of gastrointestinal tumors.^28^ In the same study, they showed that depletion of PKD2 in pancreatic tumors inhibited tumor driven blood vessel formation in a model for angiogenesis, as well as in orthotopic pancreatic cancer xenografts. Interestingly, mice lacking PKD1 die *in utero*, whilst mice lacking PKD2 are phenotypically normal and viable, indicating that PKD2 is not strictly required for the functioning of normal cells.^30^ These combined studies indicate that PKD2 is a crucial mediator of the angiogenic response being both needed in the tumor endothelial cell (for proliferation, migration and tube formation) as well as in the tumor cell (for expression and secretion of angiogenic factors). Therefore, PKD2 is an interesting target kinase to inhibit tumor development and aggressiveness. Several PKD inhibitors have recently been described by various authors, both ATP-competitive active site, and non-competitive, presumably allosteric site inhibitors. Some noteworthy examples are: an indolocarbazole Gö6976 (ref. 31) (a dual PKC/PKD inhibitor), a benzoxoloazepinolone CID755673,^32^ 2,6-naphthyridine and pyridyl inhibitors,^33^,^34^ 3,5-diarylazoles^35^ and a pyrazine benzamide.^36^

**Fig. 1:**
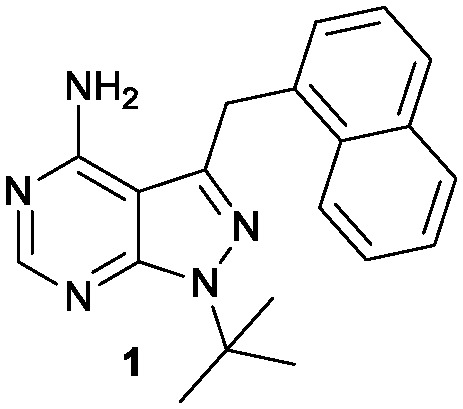
1-NM-PP1 compound.

Herein we describe the exploration of a novel PKD inhibitory chemotype based on 1-naphtylmethyl-pyrazolo[3,4-*d*]pyrimidine (1-NM-PP1, Fig. 1). 1-NM-PP1 is a bulky analog of the Src inhibitor PP1. It was originally designed to be active only towards kinases where the large so-called ‘gatekeeper’ residue is substituted with a smaller amino-acid, such as alanine, *via* site-directed mutagenesis.^37^ The pyrazolo[3,4-*d*]pyrimidine scaffold was also applied in ‘bumped’ kinase inhibitors of CDPKs found in plants and ciliates but claimed not to be active in humans.^38^ In our search for PKD2 inhibitors, we screened the Calbiochem kinase inhibitor library, during which we identified 1-NM-PP1 as a potent PKD inhibitor. Other reports also identified this compound as a lead inhibitor in a PKD screening.^39^

**Fig. 2:**
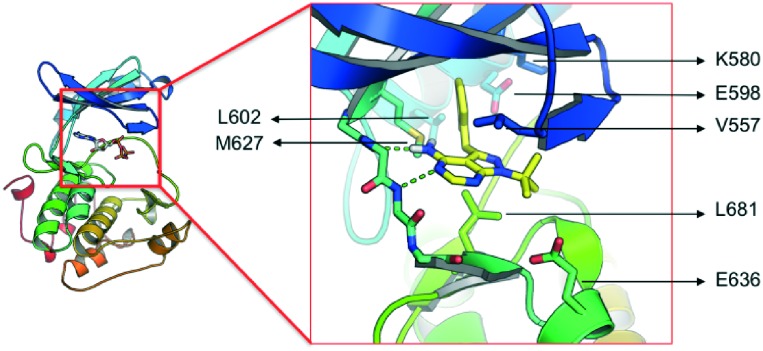
Model of the kinase domain of PKD2 with a blow up of 1-NM-PP1 bound into the ATP binding site.

The lack of activity of 1-NM-PP1 against the majority of the kinome, combined with its activity against PKD, offers some opportunities to develop a compound with selectivity towards the PKD family. Hence, we considered this compound a valid starting point to create analogues, and for exploring the SAR aiming to design a highly specific PKD inhibitor with pharmaceutical potential. To guide the design, a structural model of 1-NM-PP1 inside the PKD2 kinase domain was constructed using MOE (CCG, Montreal, Canada) employing PDB 3PFQ (PKC), ; 1KOA (Twitchin Kinase fragment) and ; 3NOY (IspG), which were identified by Phyre2 as suitable templates for modeling the PKD2 kinase domain.^40^ In this first model, 1-NM-PP1 cannot be accommodated in its typical binding mode, similar to its natural ligand ATP, as the naphthalene group clashes with the gatekeeper residue M627. Therefore, the commonly observed binding mode of 1-NM-PP1 was enforced in the binding site according to the interactions and geometry observed in PDBs ; 3NCG and ; 3I7B.^38^,^41^ In this model, shown in Fig. 2, a cavity aside from gatekeeper residue Met-627 and flanked by residues K580, E598 and V625 was introduced that accommodates the naphthalene group. From this second model it appeared that modification of this naphthalene group at the 3-position of 1-NM-PP1 could lead to improved potency and specificity. This hypothesis urged us to start modifying the 3-position and to initiate a SAR study, *via* possible modifications such as varying the aromatic substituent at the 3-position and creation of longer chain lengths in between the core scaffold and the aromatic substituent.

## Results and discussion

**Fig. 3:**
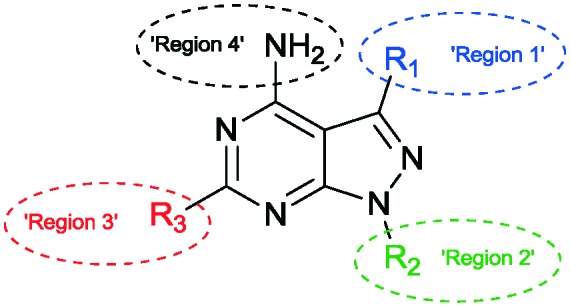
Interesting regions in the SAR study of the pyrazolo[3,4-*d*]pyrimidine based inhibitors.

The SAR study contains variations in 3 different regions as depicted in Fig. 3. Region 4 should remain untouched due to clashes with the gatekeeper when the free amine is functionalized.

**Scheme 1:**
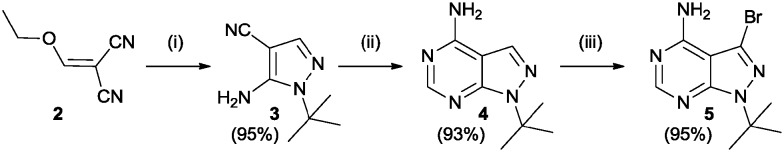
Synthesis of bromo precursor **5**, based on Todorovic *et al.* Reagents and conditions: (i) *tert*-butylhydrazine hydrochloride, Et_3_N, EtOH (ii) formamide, 150 °C (iii) Br_2_, H_2_O.^42^

The study began with the variation of region 1(R_1_). For this the 3-position of the core pyrazolo[3,4-*d*]pyrimidine scaffold was varied using different aromatic moieties. Direct functionalization was achieved based on a synthesis by Todorovic *et al.* and is depicted in Scheme 1.^42^ Starting from (ethoxymethylene)malononitrile **2** a first cyclisation was performed using *tert*-butylhydrazine hydrochloride to obtain pyrazole **3**. A second cyclisation was performed using formamide to obtain the core scaffold **4**. A bromination in aqueous environment provided compound **5**.

**Scheme 2:**
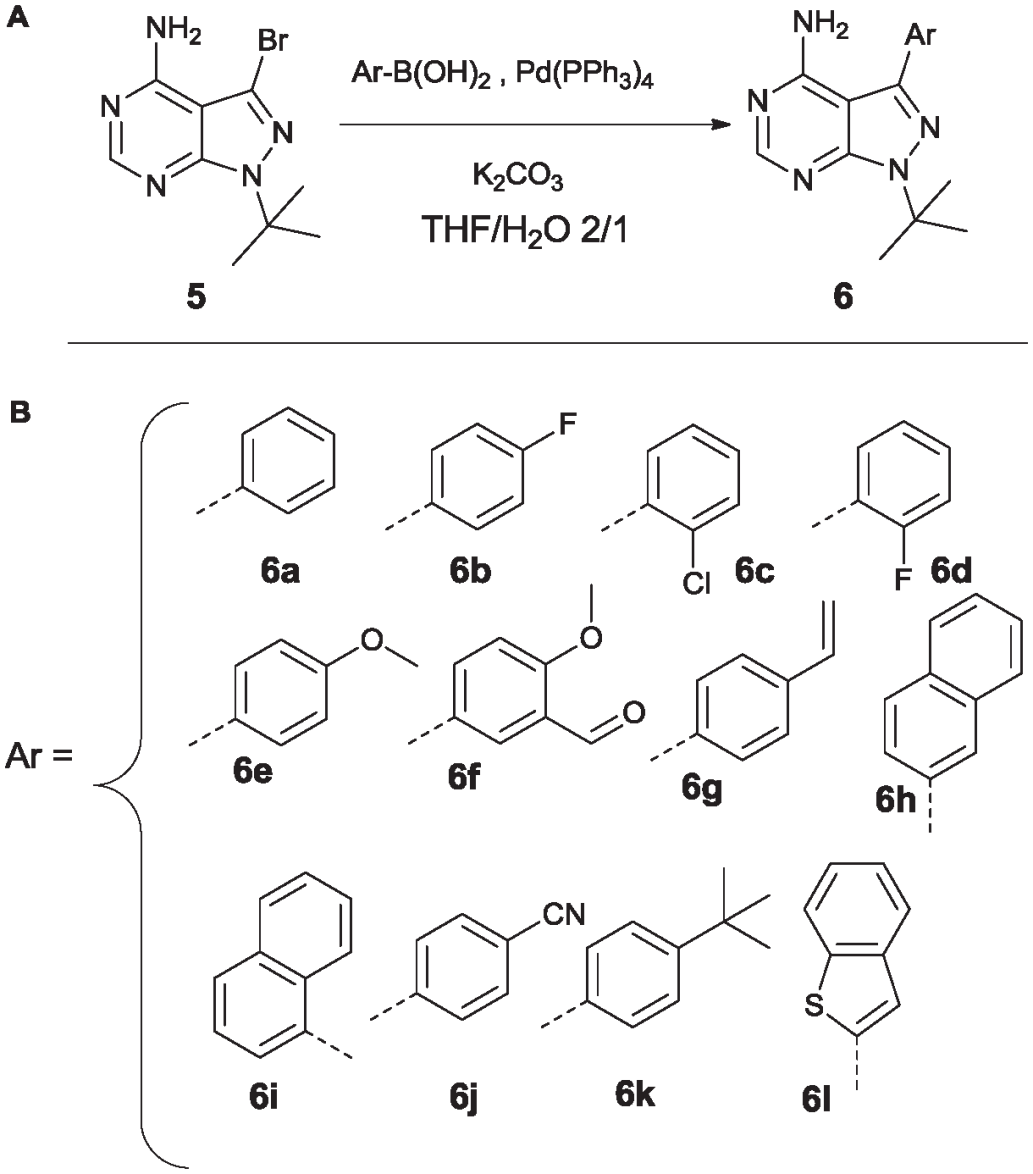
Suzuki reaction conditions to obtain variation at the 3-position (A). List of all variations synthesized (B).

Variation at the 3-position was achieved by performing Suzuki-coupling reactions with compound **5** as is depicted in Scheme 2. 12 compounds shown in Scheme 2B were tested for activity against PKD2.

**Scheme 3:**
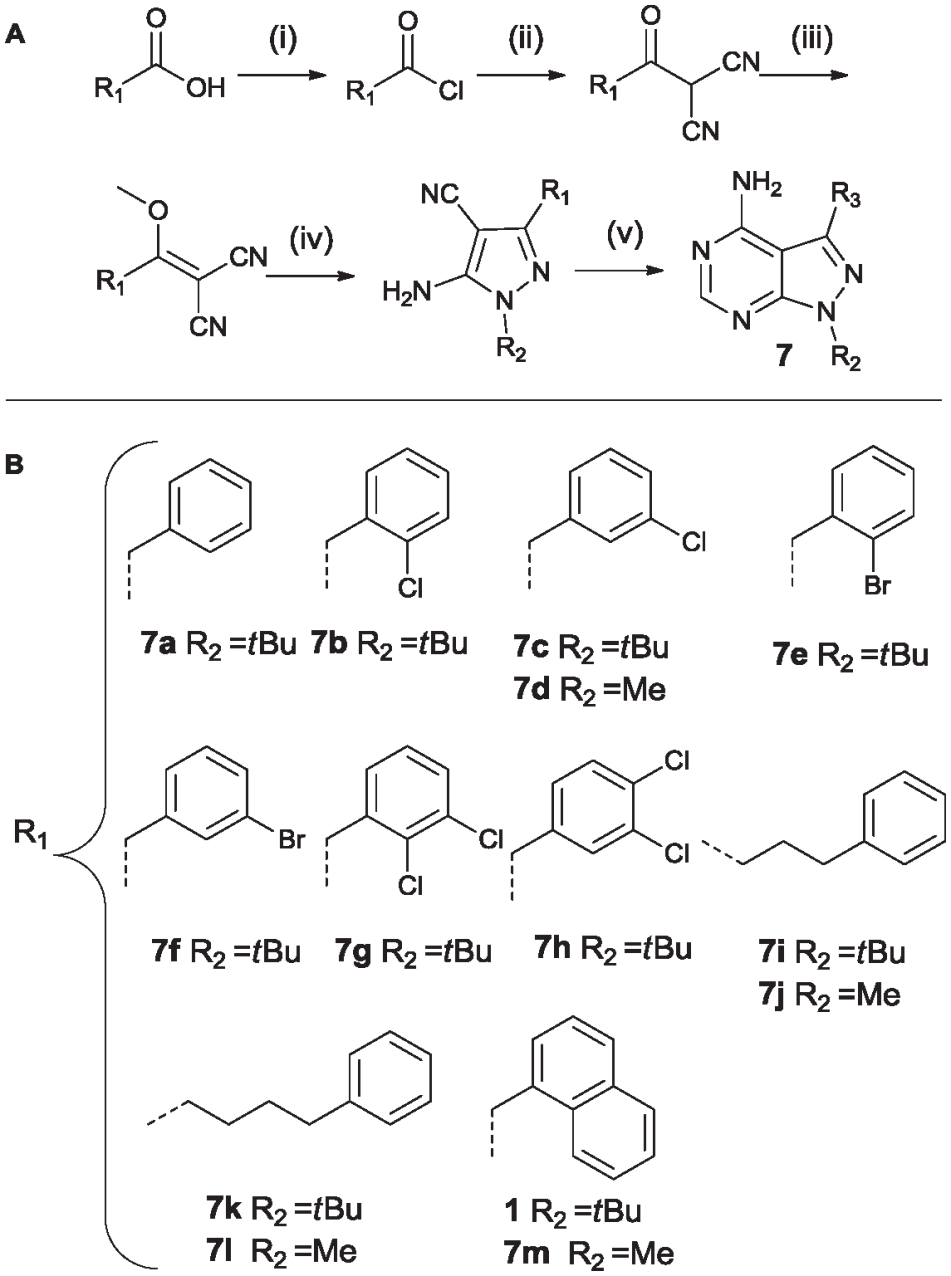
Synthesis based on Bishop *et al.* Reagents and conditions: (i) SOCl_2_, reflux (ii) malononitrile, NaH, dry THF (iii) Me_2_SO_4_, NaHCO_3_, dioxane/H_2_O (iv) *tert*-butyl/methylhydrazine hydrochloride, Et_3_N, EtOH (v) formamide, 150 °C (A).^37^ List of all variations synthesized (B).

Analogues containing a methylene linker between the core scaffold and the aromatic moeity at the 3-position was based on a procedure by Bishop *et al.* as depicted in Scheme 3.^37^ The synthetic procedure is more elaborate as the functionality is introduced at the very start of the synthesis. The acid is converted to the acid chloride and then reacted with malononitrile under basic conditions. Methylation of the intermediate followed by treatment with the appropriate hydrazine provides the pyrazole intermediate. The last step is the formation of the pyrimidine ring system using formamide at 150 °C. Region 2 was varied using methylhydrazine instead of *tert*-butylhydrazine (compounds **7d**, **7j**, **7l**, **7m**).

**Scheme 4:**
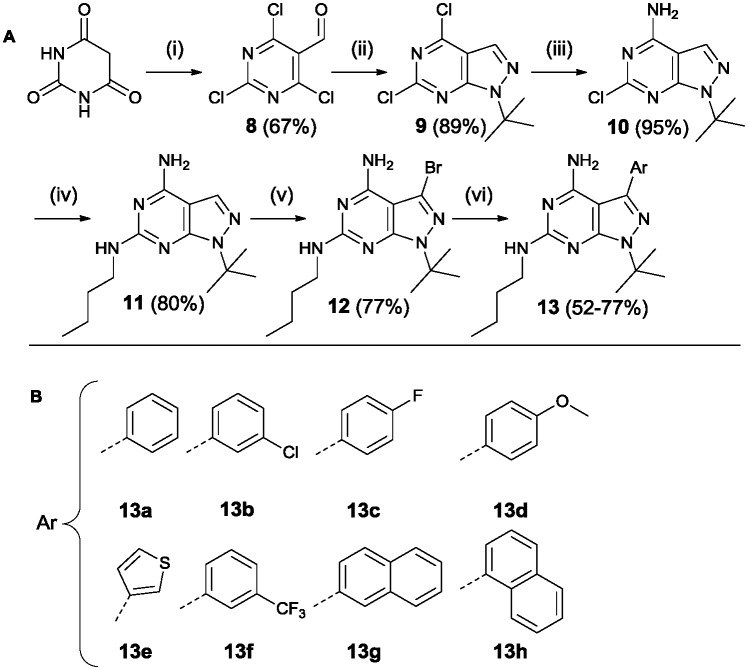
Synthesis based on Verheijen *et al.* Reagents and conditions: (i) POCl_3_, DMF (ii) *tert*-butyl hydrazine hydrochloride, Et_3_N, MeOH (iii) 25% aq. NH_4_OH, dioxane (iv) *n*-butylamine (v) Br_2_, DCM (vi) Pd(PPh_3_)_4_, K_2_CO_3_, THF/H_2_O 2/1 (A).^43^ List of all variations synthesized (B).

Region 3 was varied using a third synthetic approach to introduce variations at the 6-position. Based on the results from molecular modelling, it was hypothesized that an extra interaction (hydrogen donor) with the hinge region could increase compound activity. For this reason a synthetic approach was used based on a procedure by Verheijen *et al.* depicted in Scheme 4.^43^ Starting from barbituric acid, a Vilsmeier–Haack reaction provided 2,4,6-trichloro-5-formyl-pyrimidine **8**. Cyclisation with *tert*-butyl hydrazine hydrochloride yielded compound **9**. The latter was used in two subsequent aromatic substitution reactions with ammonium hydroxide and *n*-butylamine, respectively. Bromination provided the starting compound **12** which was further functionalized using Suzuki coupling reactions.

**Scheme 5:**
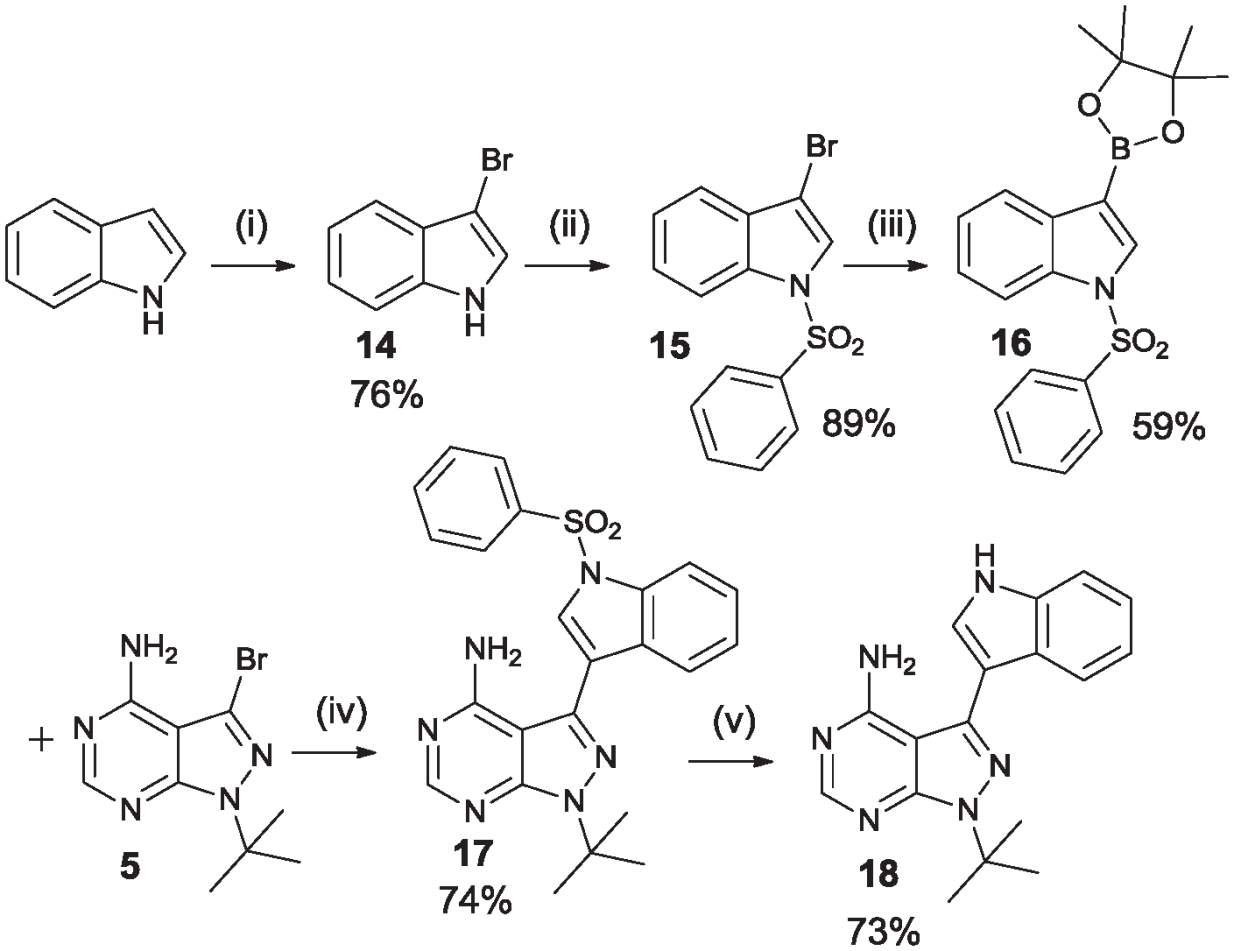
Synthethic procedure of the 3-IN-PP1 compound. Reagents and conditions: (i) pyridinium tribromide, pyridine, rt (ii) benzenesulfonyl chloride, TBAHS, 50% aq-NaOH, toluene, rt (iii) bis(pinacolato)diboron, Pd(dppf)Cl_2_, KOAc, DMF, 80 °C (iv) Pd(PPh_3_)_2_Cl_2_, Na_2_CO_3_, dioxane/H_2_O 4/1, reflux (v) TBAF, THF, reflux.

Another final synthetic procedure was to verify if adding an extra hydrogen acceptor functionality in the aromatic moiety at the 3-position would improve potency. Scheme 5 shows the synthesis of the 3-IN-PP1 (**18**), containing an NH hydrogen donor in the form of an indole. Starting from the commercially available indole, bromination at the 3-position was achieved using pyridinium tribromide in pyridine. Protection of the free NH-functionality with benzenesulfonyl chloride and formation of the boronate ester with bis(pinacolato)diboron followed by Suzuki coupling and deprotection of the indole yielded the desired product 3-indolylmethyl-pyrazolo[3,4-*d*]pyrimidine (3-IN-PP1).

All 35 described analogues were characterized *in vitro via* an initial screening at 1 μM against PKD2. This revealed some unexpected properties (for complete data see ESI†). Based on the results of the initial screening, a subset of compounds was selected in order to construct a structure–activity relationship (SAR) based on IC_50_ values (see Table 1 for IC_50_ values).

**Table 1:** Residual PKD2 activity at 1 μM and corresponding IC_50_ values in μM shown for 1-NM-PP1 and 13 analogues

Compound	% PKD 2 activity remaining (1 μM)	IC_50_ (μM)
	(Average ± SD)	
**1-NM-PP1** (**1**)	23 ± 1.7	0.398
**7a**	35 ± 1.3	0.449
**1-NA-PP1** (**6i**)	34 ± 0.4	0.403
**7b**	41 ± 2.6	0.451
**7i**	101 ± 8.3	>50
**7c**	55 ± 2.4	0.814
**7d**	83 ± 6.4	4.841
**7e**	43 ± 6.6	0.535
**6a**	76 ± 2.9	9.488
**7k**	93 ± 5.8	>50
**7m**	64 ± 3.9	4.657
**13h**	89 ± 6.2	8.169
**3-IN-PP1** (**18**)	8 ± 0.8	0.033
**6l**	53 ± 7.1	2.206

**Fig. 4:**
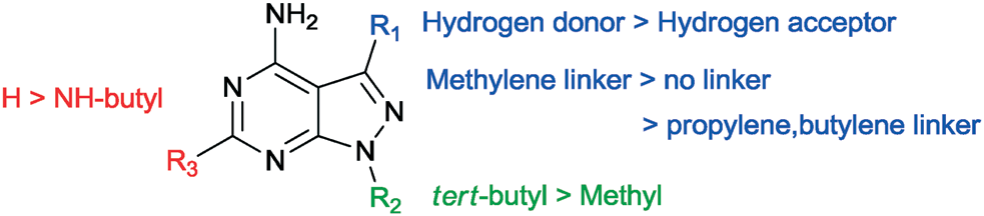
Summary of the SAR for the provided compounds.

In our SAR study, 1-NM-PP1 is used as a reference to compare these results with (**1**, IC_50_ of 0.398 μM). The IC_50_ we observed is higher than previously reported by the Wang group (IC_50_ of 0.133 μM). This is likely due to differences in assay setup and differences in enzyme preparation (expression system and activation of PKD, see ESI† for more information). When we change the substituent on the 3-position in region 1 (Fig. 3) of the pyrazolo[3,4-*d*]pyrimidine scaffold to a benzyl group (**7a**, IC_50_ of 0.449 μM), activity is slightly decreased compared to 1-NM-PP1. Deletion of the benzylic carbon resulted in a similar inhibition towards PKD2 for **6i** (IC_50_ of 0.403 μM). Follow-up modification of the phenyl group with electron donating and electron withdrawing groups slightly reduced activity (*e.g.***7b**, IC_50_ of 0.451 μM). Further chain elongation of the methylene linker completely abolished activity (**7i**, IC_50_ of >50 μM). *Via* these analogues it was established that an optimum is reached when using benzylic substituents at the 3-position. The *N*-substituent in region 2 was also varied between *tert*-butyl and methyl (**7c**, **7d**, **7i**, **7j**, **7k**, **7l**, **1**, **7m**). These results along with the paper from Tandon *et al.*^39^ suggest that a *tert*-butyl group seemed to be most suitable for this position, since we observed a loss in potency upon changing a *tert*-butyl (**7c**, IC_50_ of 0.814 μM) to a methyl group (**7d**, IC_50_ of 4.841 μM). It was also hypothesized that the addition of an extra NH-functionality could provide an extra hydrogen acceptor to interact with the hinge region of PKD. For this reason *n*-butyl amine was added at the 6-position of the pyrazolo[3,4-*d*]pyrimidine scaffold. The long alkylchain should not hinder due to it pointing towards the outside of the binding pocket. However these compounds displayed strongly reduced activity (**13h**, IC_50_ of 8.169 μM) when compared to 1-NM-PP1. Although all previous analogues did not result in an increase in potency when compared to 1-NM-PP1, compound **18** (3-IN-PP1) showed strong PKD2 inhibition with an IC_50_ of 0.033 μM, or about a 10 fold increase in potency compared to 1-NM-PP1. Thus the addition of a hydrogen donor at the 3-substituent seems to increase potency by a substantial amount. The addition of a hydrophobic benzothiophene on the other hand seemed to reduce activity (**6l**, IC_50_ of 2.206 μM). The findings described above are summarized in Fig. 4.

**Fig. 5:**
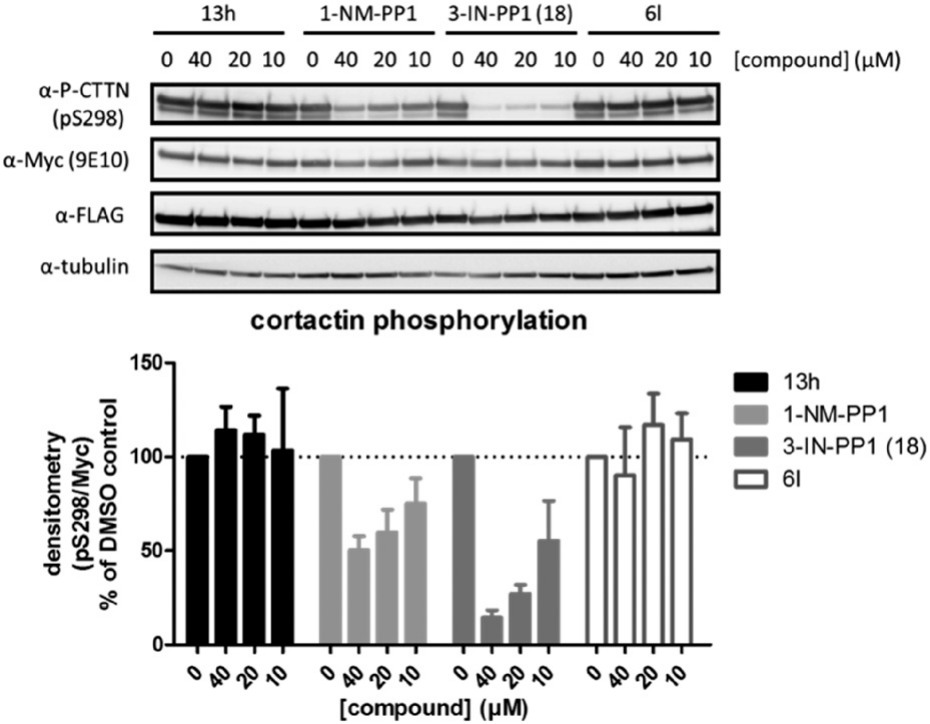
The effect of 1-NM-PP1 analogues on PKD mediated phosphorylation of cortactin at Ser-298. HEK293T cells co-transfected with FLAG-PRKD2 and myc-CTTN were pretreated with the indicated compounds for 30 min prior to stimulation with 500 nM Phorbol 12,13-dibutyrate (PDB) for 15 min. Phosphorylation of cortactin was followed *via* western blot using a home-made pSer-298 antibody and subsequently reprobed for total cortactin (anti-c-Myc ; 9E10) and PKD (anti-FLAG M2). Quantification of pSer-298/c-Myc signals of three independent experiments is shown in the graph below.

We also investigated if these compounds, especially 3-IN-PP1 (**18**), also showed similar activity profiles in a cellular setting. Therefore we used a cellular assay in which we were able to specifically follow PKD mediated phosphorylation of cortactin, a known PKD substrate, at Ser-298.^15^ Probing for substrate phosphorylation has advantages compared to other readouts used for PKD activity such as C-terminal auto-phosphorylation, which has been shown to be a very efficient event, quite resistant to inhibition.^44^ Activation loop Ser-738/742 phosphorylation is also an unfavorable readout for PKD activity, since it has been shown to be unexpectedly upregulated upon inhibition.^45^ Cortactin phosphorylation at Ser-298 in our assay was PKD-specific, since co-transfection of PKD and cortactin was necessary to produce an adequate signal (data not shown). We selected four compounds for further validation in a cellular assay against PKD2 (Fig. 5). Based on *in silico* predictions, **13h** was expected to show increased potency compared to 1-NM-PP1 due to its extra interaction with the hinge region, but this was not the case (IC_50_ of 8.169 μM) which is supported by its negligible cellular activity. Both 1-NM-PP1 and 3-IN-PP1 (**18**) display dose-dependent inhibition of PKD2 in our cellular system. As is mirrored in our *in vitro* assays, 3-IN-PP1 (**18**) showed highly increased potency compared to 1-NM-PP1, with almost full inhibition at 40 μM (85% inhibition compared to 50% for 1-NM-PP1). In contrast, compound **6l**, containing a more hydrophobic benzothiophene, did not show cellular activity as was expected from its *in vitro* potency.

**Fig. 6:**
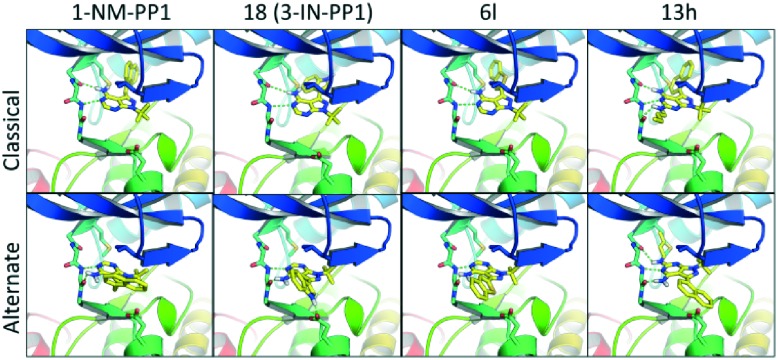
The top row represents the binding mode of representative compounds according to the binding mode of 1-NM-PP1 as observed in classic kinase structures. The bottom row represents an alternate secondary binding mode which is in better agreement with the observed SAR.

Anticipated from our initial modelling results, the analogues showed some unexpected results. Docking a diverse set of structurally different analogues (**1**, **18**, **6l**, **13h** shown in Fig. 6) to the first PKD2 model, without the binding mode of 1-NM-PP1 enforced, revealed a predominant binding mode different from the commonly observed binding mode where the pyrazolo[3,4-*d*]pyrimidine compounds are bound in a similar fashion when compared to PKD's natural ligand ATP. In this alternate binding mode, the pyrazolo[3,4-*d*]pyrimidine compound is flipped 180 degrees where the gatekeeper residue does not require an induced fit to accommodate the phenyl or benzyl substituent (Fig. 6). This alternate binding mode is in better agreement with the observed SAR and explains the lack of influence on the activity by the different decorations on the now solvent exposed phenyl and benzyl substituents. It also explains the 10-fold increase in activity of 3-IN-PP1 (**18**), since the NH-hydrogen is stabilized by a Glu636 residue in the alternate binding mode as depicted in Fig. 6. For this reason **6l** is destabilized since its free electron pair of the sulfur atom clashes with this hydrophilic environment. Compound **6l** was expected to be more potent in the classical binding mode, since the 3-position points towards a hydrophobic pocket, supporting the hypothesis of this alternate binding mode. Also the complete lack of activity of **13h** can be explained as the butyl tail now clashes with the gatekeeper residue, while it is completely solvent exposed in the first binding mode. All crystal structures of 1-NM-PP1 derivatives (PDBs ; 2WEI, ; 3I7B, ; 3MWU, ; 3N51, ; 3NCG, ; 3SVV, ; 3MA6, ; 4LGG, ; 4QOX) exhibit an identical binding mode for the purine ring isostere in protein kinase structures in accordance with the binding mode of ATP. Interestingly, this inversed alternate binding mode is already observed in one structure (PDB ; 4GKI).^46^ While this protein is not a protein kinase, but an aminoglycoside phosphotransferase, the overall protein fold is identical to the protein kinase active domain architecture. This supports the possibility of the proposed alternate binding mode. Further exploration of region 2 and 4 is now highly recommended, since the free amine in the alternate binding mode is not blocked by the gatekeeper residue and the alkyl substituent at the 1-position now points towards a hydrophobic pocket. In addition, earlier described ‘bumped’ kinase inhibitors (BKI's) may need to take PKD side effects into account.^38^,^47^

## Conclusions

In summary, we rationally explored the pyrazolo[3,4-*d*]pyrimidine structure to find potential novel PKD2 inhibitors with enhanced inhibitory properties. We identified 3-IN-PP1 as a potent PKD inhibitor, displaying a 10-fold increase in *in vitro* potency (IC_50_ of ±33 nM) when compared to 1-NM-PP1. In combination with its remarkable *in cellulo* potency, 3-IN-PP1 is a very promising target for further optimization studies. This compound along with other pyrazolo[3,4-*d*]pyrimidine analogues studied in this paper, exposed some interesting trends as shown in Scheme 5. These trends point towards an alternate binding mode where the pyrazolo[3,4-*d*]pyrimidine is flipped 180 degrees in comparison with the normal binding mode (Fig. 6). This new binding mode for pyrazolo[3,4-*d*]pyrimidine compounds means that further structure based optimization may open up a completely new class of specific kinase inhibitors for kinases that show sensitivity towards pyrazolo[3,4-*d*]pyrimidine derived inhibitors.

## Supplementary Material

Supplementary information
