# Supplementary material for: Discovery of a potent protein kinase D inhibitor: insights in the binding mode of pyrazolo[3,4-d]pyrimidine analogues
Source: Medchemcomm. 2017 Feb 9;8(3):640–6. doi: 10.1039/c6md00675b (PMC5567267; doi:10.1039/c6md00675b)
Supplement: Supplementary file 1 [file MD-008-C6MD00675B-s001.pdf]

Electronic Supplementary Material (ESI) for MedChemComm.
This journal is © The Royal Society of Chemistry 2017

Discovery of a potent protein kinase D inhibitor: insights in the binding mode of pyrazolo[3,4-*d*]pyrimidine analogues.

Verschueren Klaas[a†], Mathias Cobbaut[b†], Joachim Demaerel[a], Lina Saadah[b], Arnout R.D. Voet[c], Johan Van Lint[b] and Wim M. De Borggraeve[a*]

[a]Department of Chemistry, Molecular Design and Synthesis, KU Leuven, Celestijnenlaan 200F, 3001 Leuven, Belgium
[b]Department of Cellular and Molecular Medicine, Laboratory of Protein Phosphorylation and Proteomics, KU Leuven, Herestraat 49 box 901, 3000 Leuven, Belgium
[c]Department of Chemistry, Laboratory of Biomolecular Modeling and Design, KU Leuven, Celestijnenlaan 200G, 3001 Leuven, Belgium
[†]These authors contributed equally to this work

* To whom correspondence should be addressed.
Tel: +32 16 32 76 93
Email: wim.deborggraeve@kuleuven.be

Content

Chemicals and Reagents 3
Synthesis 3
(ethoxymethylene)malononitrile (**2**) 3
5-amino-1-(tert-butyl)-1H-pyrazole-4-carbonitrile (**3**) 3
1-(tert-butyl)-1H-pyrazolo[3,4-d]pyrimidin-4-amine (**4**) 3
3-bromo-1-(tert-butyl)-1H-pyrazolo[3,4-d]pyrimidin-4-amine (**5**) 4
General procedure A for the Suzuki-Miyaura cross-coupling reaction 4
1-tert-butyl-3-phenyl-1H-pyrazolo[3,4-d]pyrimidin-4-amine (**6a**) 4
1-tert-butyl-3-(4-fluorophenyl)-1H-pyrazolo[3,4-d]pyrimidin-4-amine (**6b**) 4
1-tert-butyl-3-(2-chlorophenyl)-1H-pyrazolo[3,4-d]pyrimidin-4-amine (**6c**) 4
1-tert-butyl-3-(2-fluorophenyl)-1H-pyrazolo[3,4-d]pyrimidin-4-amine (**6d**) 5
1-tert-butyl-3-(4-methoxyphenyl)-1H-pyrazolo[3,4-d]pyrimidin-4-amine (**6e**) 5
2-acetyl-5-(4-amino-1-(tert-butyl)-1H-pyrazolo[3,4-d]pyrimidin-3-yl)benzaldehyde (**6f**) 5
1-(tert-butyl)-3-(4-vinylphenyl)-1H-pyrazolo[3,4-d]pyrimidin-4-amine (**6g**) 5
1-tert-butyl-3-(2-naphthyl)-1H-pyrazolo[3,4-d]pyrimidin-4-amine (**6h**) 5
1-tert-butyl-3-(1-naphthyl)-1H-pyrazolo[3,4-d]pyrimidin-4-amine (**6i**) 5
4-(4-amino-1-(tert-butyl)-1H-pyrazolo[3,4-d]pyrimidin-3-yl)benzonitrile (**6j**) 5
1-(tert-butyl)-3-(4-(tert-butyl)phenyl)-1H-pyrazolo[3,4-d]pyrimidin-4-amine (**6k**) 6
General procedure B for the formation of the pyrimidine-ring system 6
3-benzyl-1-tert-butyl-1H-pyrazolo[3,4-d]pyrimidin-4-amine (**7a**) 6
1-tert-butyl-3-(2-chlorobenzyl)-1H-pyrazolo[3,4-d]pyrimidin-4-amine (**7b**) 6

1-tert-butyl-3-(3-chlorobenzyl)-1H-pyrazolo[3,4-d]pyrimidin-4-amine (**7c**) 6
3-(3-chlorobenzyl)-1-methyl-1H-pyrazolo[3,4-d]pyrimidin-4-amine (**7d**) 7
3-(2-bromobenzyl)-1-tert-butyl-1H-pyrazolo[3,4-d]pyrimidin-4-amine (**7e**) 7
3-(3-bromobenzyl)-1-tert-butyl-1H-pyrazolo[3,4-d]pyrimidin-4-amine (**7f**) 7
1-tert-butyl-3-(2,3-dichlorobenzyl)-1H-pyrazolo[3,4-d]pyrimidin-4-amine (**7g**) 7
1-tert-butyl-3-(3,4-dichlorobenzyl)-1H-pyrazolo[3,4-d]pyrimidin-4-amine (**7h**) 7
1-tert-butyl-3-(3-phenylpropyl)-1H-pyrazolo[3,4-d]pyrimidin-4-amine (**7i**) 7
1-methyl-3-(3-phenylpropyl)-1H-pyrazolo[3,4-d]pyrimidin-4-amine (**7j**) 7
1-tert-butyl-3-(4-phenylbutyl)-1H-pyrazolo[3,4-d]pyrimidin-4-amine (**7k**) 8
1-methyl-3-(4-phenylbutyl)-1H-pyrazolo[3,4-d]pyrimidin-4-amine (**7l**)..8
1-tert-butyl-3-(1-naphthylmethyl)-1H-pyrazolo[3,4-d]pyrimidin-4-amine (**1**) 8
1-methyl-3-(1-naphthylmethyl)-1H-pyrazolo[3,4-d]pyrimidin-4-amine (**7m**) 8
2,4,6-trichloropyrimidine-5-carbaldehyde (**8**) 8
1-tert-butyl-4,6-dichloro-1H-pyrazolo[3,4-d]pyrimidine (**9**) 8
1-tert-butyl-6-chloro-1H-pyrazolo[3,4-d]pyrimidin-4-amine (**10**) 9
N6-butyl-1-tert-butyl-1H-pyrazolo[3,4-d]pyrimidine-4,6-diamine (**11**) 9
3-bromo-N6-butyl-1-tert-butyl-1H-pyrazolo[3,4-d]pyrimidine-4,6-diamine (**12**) 9
N6-butyl-1-tert-butyl-3-phenyl-1H-pyrazolo[3,4-d]pyrimidine-4,6-diamine (**13a**) 9
N6-butyl-1-tert-butyl-3-(3-chlorophenyl)-1H-pyrazolo[3,4-d]pyrimidine-4,6-diamine (**13b**)............10
N6-butyl-1-tert-butyl-3-(4-fluorophenyl)-1H-pyrazolo[3,4-d]pyrimidine-4,6-diamine (**13c**).............10
N6-butyl-1-tert-butyl-3-(4-methoxyphenyl)-1H-pyrazolo[3,4-d]pyrimidine-4,6-diamine (**13d**)........10
N6-butyl-1-tert-butyl-3-(3-thienyl)-1H-pyrazolo[3,4-d]pyrimidine-4,6-diamine (**13e**) 10
N6-butyl-1-tert-butyl-3-[3-(trifluoromethyl)phenyl]-1H-pyrazolo[3,4-d]pyrimidine-4,6-diamine (**13f**) 10
N6-butyl-1-tert-butyl-3-(2-naphthyl)-1H-pyrazolo[3,4-d]pyrimidine-4,6-diamine (**13g**) 11
N6-butyl-1-tert-butyl-3-(1-naphthyl)-1H-pyrazolo[3,4-d]pyrimidine-4,6-diamine (**13h**)...................11
3-bromo-1H-indole (**14**) 11
3-bromo-1-(phenylsulfonyl)-1H-indole (**15**) 11
1-(phenylsulfonyl)-3-(4,4,5,5-tetramethyl-1,3,2-dioxaborolan-2-yl)-1H-indole (**16**) 11
1-tert-butyl-3-[1-(phenylsulfonyl)-1H-indol-3-yl]-1H-pyrazolo[3,4-d]pyrimidin-4-amine (**17**) 12
1-tert-butyl-3-(1H-indol-3-yl)-1H-pyrazolo[3,4-d]pyrimidin-4-amine (**18**)......................................12
Production and purification of GST-PKD1/2 in HEK293T cells 12
PKD2 screening assay 13
Cellular screening assay using phospho-cortactin as a readout 13
% inhibition of PKD2 at 1µM 14
Enlarged version of figure 5 15

Chemicals and Reagents

All reagents and solvents were of analytical grade and purchased from commercial sources (Sigma-Aldrich, Acros, TCI, Strem, Fluka) and were used without further purification. ^{1}H NMR spectra were recorded on a Bruker Avance 300 or Bruker Avance 400 spectrometer. Chemical shifts are reported in parts per million (ppm) and are referenced to the internal standard tetramethylsilane (TMS). Thin Layer Chromatography (TLC) was performed using 0,25mm thick pre-coated TLC plates SIL G-25 (with fluorescence indicator at 254nm) visualized under a UV lamp at 254 nm. Column chromatography was carried out using flash silica (Davisil®Chromatography Silica Medium type LC 60A, 40-63micron) on a Büchi SepacoreTM flash apparatus, consisting of a C-660 Büchi fraction collector, C-615 Pump manager, C-635 UV-photometer, two C-605 pump modules and a Linseis D120S plotter. EI-MS data were recorded by a Hewlett Packard 5989A Quadrupole equipment linked to a HP Apollo 900 series 400 data processing system. GCMS measurements were performed with a ThermoFinnigan Trace™ GC Gas chromatographer with non-automated PTV injector, temperature program from 40°C to 300°C (rate:20°C/min, hold 5 min), coupled to a ThermoScientific ITQ900 full scan EI mass spectrometry detector.

Synthesis

*(ethoxymethylene)malononitrile (**2**)*

A 100 mL flask was loaded with triethylorthoformate (13 ml, 76 mmol) and malononitrile (5 g, 76 mmol) in Ac_2O (16 mL). The reaction mixture was refluxed overnight. Next the solvent was removed *in vacuo*. The residue was purified using MPLC (Hept/EtOAc 1/1 v/v) to yield 7.6 g of the title compound (82%). ^{1}H-NMR (600 MHz, $CDCl_3$) δ 7.68 (s, 1H), 4.39 (q, J = 7.15 Hz, 2H), 1.42 (d, J = 7.15 Hz, 3H). ^{13}C-NMR (75 MHz, $CDCl_3$) δ 174.78, 112.27, 110.14, 75.20, 66.49, 15.10. EI-MS (m/z): 122 $[M]^+$.

*5-amino-1-(tert-butyl)-1H-pyrazole-4-carbonitrile (**3**)*

Tert-butylhydrazine hydrochloride (3.40 g, 27.3 mmol) and triethylamine (2.76 g, 27.3 mmol) were added to a two-necked flask in anhydrous EtOH (425 mL). 2-(ethoxymethylene)malononitrile (**2**) (5.000 g, 40.9 mmol) was slowly added in portions. The reaction mixture was heated at reflux for 3hr. The crude product was concentrated *in vacuo* and the residue was purified using MPLC (95/5, DCM/MeOH, v/v) to yield the product as an orange solid (95%). ^{1}H-NMR (300 MHz, $CDCl_3$,):δ8.36 (s, 1H), 7.86 (s, 1H), 5.91 (bs, 2H), 1.80 (s, 9H). ^{13}C-NMR (75 MHz, $CDCl_3$): δ149.8, 138.3, 114.4, 78.3, 60.0, 29.0 EI-MS (m/z): 165 $[M]^+$. HRMS calculated mass 164.10620; found mass 164.10483.

*1-(tert-butyl)-1H-pyrazolo[3,4-d]pyrimidin-4-amine (**4**)*

5-amino-1-(*tert*-butyl)-1*H*-pyrazole-4-carbonitrile (**3**) (0.200 g, 1.218 mmol) and formamide (1.937 mL, 48.7 mmol) were added to a pressure tube. The reaction mixture was heated at 150°C for 12hr. After cooling down to room temperature the reaction mixture was extracted 3 times with EtOAc. The combined organic layers were washed respectively with a saturated $NaHCO_3$ solution, water and brine. The organic layer was dried with $MgSO_4$, filtered and concentrated *in vacuo*. The crude product was purified using MPLC (95/5 DCM/MeOH v/v) to yield the product as a pale white solid (93%). ^{1}H-NMR (300 MHz, $CDCl_3$, ppm): δ8.30 (s, 1H), 6.39 (b,

2H), 1.77 (s, 9H). ^{13}C-NMR (75 MHz, $CDCl_3$, ppm): δ157.6, 154.4, 153.2, 128.5, 102.0, 60.4, 29.1. EI-MS (m/z): 191 $[M]^+$. HRMS calculated mass 191.11710; found mass 191.11463.

*3-bromo-1-(tert-butyl)-1H-pyrazolo[3,4-d]pyrimidin-4-amine (**5**)*

1-(*tert*-butyl)-1*H*-pyrazolo[3,4-*d*]pyrimidin-4-amine (**4**) (1.000 g, 5.23 mmol) was dissolved in water (40 mL), stirred and cooled to 0°C in an ice bath. Bromine (0.539 mL, 10.46 mmol) was added and the reaction mixture was stirred at room temperature for 1hr before refluxing for 1hr. The reaction mixture was quenched with 10% $Na_2S_2O_3$ and extracted with EtOAc(3x). The organic layers were combined and washed with brine, dried using $MgSO_4$ and filtered after which the reaction mixture was concentrated *in vacuo* to yield the product as a pale yellow solid (95%). ^{1}H-NMR (300 MHz, $CDCl_3$): δ8.30 (s, 1H), 6.39 (b, 2H), 1.77 (s, 9H). ^{13}C-NMR (75 MHz, $CDCl_3$): δ157.5, 155.1, 153.8, 115.4, 101.4, 61.5, 29.1. EI-MS (m/z): 269-271 $[M]^+$. HRMS calculated mass 269.02761; found mass 269.02741.

General procedure A for the Suzuki-Miyaura cross-coupling reaction

A 10 mL microwave vessel was charged with an arylhalide (0.185 mmol), $Pd(PPh_3)_4$ (0.019 mmol), a boronic acid (0.278 mmol) and K_2CO_3 (0.555 mmol). The vessel was degassed using a Schlenk line(3x). Next degassed THF (2 mL) and H_2O (1mL) were added and the reaction mixture was stirred at reflux overnight. The crude product was filtered over a path of celite and rinsed with EtOAc. The filtrate was extracted with H_2O. The combined organic layers were washed with brine, dried with $MgSO_4$, filtered and concentrated *in vacuo*. The crude product was purified using MPLC (heptane/EtOAc, 8/2, v/v) to obtain the desired product.

*1-tert-butyl-3-phenyl-1H-pyrazolo[3,4-d]pyrimidin-4-amine (**6a**)*

Compound **6a** was prepared using general procedure **A** (70%). ^{1}H-NMR (300 MHz, $CDCl_3$): δ 8.36 (s, 1H), 7.70 (d, J=6.6 Hz, 2H), 7.51 (m, 3H), 5.57 (bs, 2H), 1.84 (s, 9H). ^{13}C-NMR (75 MHz, $CDCl_3$): δ 157.9, 154.4, 154.2, 142.1, 133.8, 129.6, 128.8, 128.6, 99.7, 60.4, 29.2. EI-MS (m/z): 267 $[M]^+$. HRMS: Calculated mass: 286,1558; Found mass: 268,1557.

*1-tert-butyl-3-(4-fluorophenyl)-1H-pyrazolo[3,4-d]pyrimidin-4-amine (**6b**)*

Compound **6b** was prepared using general procedure A (57%). ^{1}H-NMR (300 MHz, $CDCl_3$) δ 8.36 (s, 1H), 7.68 (m, 2H), 7.23 (m, 2H), 5.45 (bs, 2H), 1.83 (s, 9H). ^{13}C-NMR (75 MHz, $CDCl_3$) δ 164.81, 157.69, 154.46, 154.22, 141.04, 130.52, 130.41, 129.86, 116.46, 116.17, 99.71, 60.54, 29.18. ^{13}C NMR (75 MHz, $CDCl_3$) δ163.2 (d, J^1= 247Hz), 157.7(s), 154.5(s), 154.2(s), 141.0(s), 130,5 (d, J^3 = 8.2Hz), 129.8 (d,^4J = 3.4Hz), 116.3 (d, J^2 = 21.7Hz), 99.7(s), 60.5(s), 29.2(s). EI-MS (m/z): 285 $[M]^+$. HRMS: Calculated mass: 286,1462; Found mass: 286,1463.

*1-tert-butyl-3-(2-chlorophenyl)-1H-pyrazolo[3,4-d]pyrimidin-4-amine (**6c**)*

Compound **6c** was prepared by using general procedure **A** (46%). ^{1}H-NMR (300 MHz, $CDCl_3$) δ 8.35 (s, 1H), 7.57 – 7.51 (m, 2H), 7.46 – 7.39 (m, 2H), 5.23 (s, 2H), 1.84 (s, 9H). ^{13}C-NMR (100 MHz, $CDCl_3$) δ 157.6, 154.5, 153.8, 139.1, 134.0, 132.5, 132.2, 130.6, 130.3, 127.3, 101.2, 60.6, 29.2. EI-MS (m/z): 302 $[M]^+$.

*1-tert-butyl-3-(2-fluorophenyl)-1H-pyrazolo[3,4-d]pyrimidin-4-amine **(6d)***

Compound **6e** was prepared by using general procedure A (64%). ^{1}H-NMR (300 MHz, $CDCl_3$) δ 8.35 (s, 1H), 7.63 - 7.57 (m, 1H), 7.48 - 7.46 (m, 1H), 7.33 – 7.22 (m, 2H), 5.40 (bs, 2H), 1.84 (s, 9H). ^{13}C-NMR (75 MHz, $CDCl_3$) δ 159.9 (d, ^1J = 269 Hz), 157.8 (s), 154.4 (s), 154.0(s), 136.0(s), 132.0 (d, ^4J = 2.6Hz), 131.0 (d, ^3J = 8.0 Hz), 125.0 (d, ^3J = 3.7 Hz), 121.5 (d, ^2J = 20.6 Hz), 116.4 (d, ^2J = 21.4 Hz), 101.2(s), 60.7(s), 29.2(s). EI-MS (m/z): 285 $[M]^+$. HRMS: Calculated mass: 286,1462; Found mass: 286,1460.

*1-tert-butyl-3-(4-methoxyphenyl)-1H-pyrazolo[3,4-d]pyrimidin-4-amine **(6e)***

Compound **6f** was prepared using general procedure A (67%). ^{1}H NMR (300 MHz, $CDCl_3$) δ 8.31 (s, 1H), 7.60 (d, J = 8.6 Hz, 2H), 7.05 (d, J = 8.6 Hz, 2H), 5.55 (bs, 2H), 3.87 (s, 3H), 1.83 (s, 9H). ^{13}C-NMR (75 MHz, $CDCl_3$) δ 160.2, 157.6, 154.0, 142.1, 130.0, 125.9, 114.7, 113.9, 99.6, 60.4, 55.4, 29.2. EI-MS (m/z): 297 $[M]^+$. HRMS: Calculated mass: 298,1662; Found mass: 298,1660.

*2-acetyl-5-(4-amino-1-(tert-butyl)-1H-pyrazolo[3,4-d]pyrimidin-3-yl)benzaldehyde **(6f)***

Compound **6l** was prepared using general procedure A (51%). ^{1}H-NMR (300 MHz, $CDCl_3$, ppm): δ 10.53 (s, 1H), 8.35 (s, 1H), 8.16 (s, 1H), 7.93 (d, J = 8.46 Hz, 1H), 7.19 (d, J = 8.46 Hz, 1H), 5.53 (bs, 2H), 4.02 (s, 3H), 1.83 (s, 9H). ^{13}C-NMR (75 MHz, $CDCl_3$) δ 189.2, 158.5, 157.9, 156.4, 151.3, 148.6, 136.1, 128.5, 121.6, 112., 103.4, 56.2, 55.7, 29.4. EI-MS (m/z): 325 $[M]^+$. HRMS: Calculated mass: 325.15574; Found mass: 325.15388.

*1-(tert-butyl)-3-(4-vinylphenyl)-1H-pyrazolo[3,4-d]pyrimidin-4-amine **(6g)***

Compound **6m** was prepared using general procedure **A** (26%). ^{1}H-NMR (300 MHz, $CDCl_3$) δ 8.35 (s, 1H), 7.68 (d, J = 8.12 Hz, 2H), 7.58 (d, J = 8.12 Hz), 6.83 (dd, J = 10.89, J = 17.60 Hz, 1H), 5.87 (d, J = 17.60 Hz, 1H), 5.51 (bs, 2H), 5.36 (d, J = 10.89 Hz, 1H), 1.84 (s, 9H). ^{13}C-NMR (75 MHz, $CDCl_3$) δ 157.7, 154.3, 154.2, 141.8, 138.0, 136,2, 133.0, 128.8, 127.0, 114.9, 99.7, 60.5, 29.2. EI-MS (m/z): 293 $[M]^+$. HRMS: Calculated mass: 293.16405; Found mass: 293.16669

*1-tert-butyl-3-(2-naphthyl)-1H-pyrazolo[3,4-d]pyrimidin-4-amine **(6h)***

Compound **6h** was prepared using general procedure A (62%). ^{1}H-NMR (300 MHz, DMSO) δ 8.27 (s, 1H), 8.21 – 8.14 (m, 1H), 8.11 – 7.96 (m, 3H), 7.87 – 7.77 (m, 1H), 7.62 – 7.55 (m, 2H), 1.79 (s, 9H). ^{13}C-NMR (75 MHz, $CDCl_3$) δ 157.9, 154.5, 154.3, 142.1, 133.5, 133.2, 131.1, 129.2, 128.2, 127.9, 127.8, 126.8, 126.8, 126.2, 100.0, 60.6, 29.2. EI-MS (m/z): 317 $[M]^+$. HRMS: Calculated mass: 317.1641; Found mass: 317.1648.

*1-tert-butyl-3-(1-naphthyl)-1H-pyrazolo[3,4-d]pyrimidin-4-amine **(6i)***

Compound **6i** was prepared using general procedure **A** (65 %). ^{1}H-NMR (300 MHz, $CDCl_3$) δ 8.35 (s, 1H), 8.03–7.85 (m, 3H), 7.72 – 7.42 (m, 4H), 5.30 (s, 2H), 1.88 (s, 9H). ^{13}C-NMR (101 MHz, $CDCl_3$) δ 157.9, 154.6, 153.9, 140.4, 134.1, 131.9, 130.6, 129.6, 128.5, 128.4, 127.1, 126.5, 125.7, 125.5. EI-MS (m/z): 317 $[M]^+$. HRMS: Calculated mass: 317.1641; Found mass: 317.1647.

*4-(4-amino-1-(tert-butyl)-1H-pyrazolo[3,4-d]pyrimidin-3-yl)benzonitrile **(6j)***

Compound **6j** was prepared using general procedure **A** (60%). ^{1}H-NMR (300 MHz, $CDCl_3$, ppm) δ 8.25 (s, 1H), 7.98 (d, J = 8.27 Hz, 2H), 7.83 (d, J = 8 Hz., 2H), 6.94 (bs, 2H), 1.75 (s, 9H). ^{13}C-NMR (75 MHz, $CDCl_3$) δ

163.5, 160.0, 159.4, 145.4, 142.9, 138.1, 134.3, 124.1, 115.9, 104.0, 65.3, 33.9. EI-MS (m/z): 292 $[M]^+$. HRMS: Calculated mass: 292.14364; Found mass: 292.14380.

*1-(tert-butyl)-3-(4-(tert-butyl)phenyl)-1H-pyrazolo[3,4-d]pyrimidin-4-amine (**6k**)*

Compound **6k** was prepared using general procedure **A** (82%). ^{1}H-NMR (300 MHz, $CDCl_3$): δ 8.35 (s, 1H), 5.58 (bs, 2H), 1.83 (s, 9H), 1.37 (s, 9H). ^{13}C-NMR (75 MHz, $CDCl_3$) δ 157.8, 154.3, 154.2, 152.0, 142.1, 130.8, 128.3, 126.3, 99.7, 60.4, 34.8, 31.3, 29.2. EI-MS (m/z): 323 $[M]^+$. HRMS: Calculated mass: 323.21100; Found mass: 323.21120.

*3-(1-benzothien-2-yl)-1-tert-butyl-1H-pyrazolo[3,4-d]pyrimidin-4-amine (**6l**)*

Compound **6l** was prepared using general procedure **A** (75%). ^{1}H-NMR (300 MHz, $CDCl_3$): δ 8.28 (s, 1H), 8.04 – 7.90 (m, 2H), 7.76 (s, 1H), 7.47 – 7.35 (m, 2H), 7.16 (s, 2H), 1.77 (s, 9H). ^{13}C-NMR (75 MHz, $CDCl_3$) δ 158.3, 154.8, 154.1, 140.0, 139.1, 135.7, 135.0, 125.0, 124.6, 124.3, 123.8, 122.3, 98.7, 60.1, 28.6. EI-MS (m/z): 3267, 323$[M]^+$.

General procedure B for the formation of the pyrimidine-ring system

Only the synthetic procedure for the final step of the synthesis is given for compound series 7. The start of the synthesis is based upon Bishop *et al.*(1999). A 10 mL pressure tube was charged with a 1-alkyl-5-amino-1*H*-pyrazole-4-carbonitrile (0.35 mmol) and formamide (24.25 mmol). The reaction was heated at 150°C overnight. After TLC showed full conversion, the reaction mixture was extracted by adding H_2O and EtOAc (3x). Next the organic layers were combined and washed with brine, dried on $MgSO_4$, filtered and concentrated *in vacuo*. The crude product was purified using MPLC (heptane/EtOAc, 1/1, v/v) to obtain the desired compound.

*3-benzyl-1-tert-butyl-1H-pyrazolo[3,4-d]pyrimidin-4-amine (7**a**)*

Compound **7a** was prepared using general procedure **B** (83%). ^{1}H-NMR (300 MHz, DMSO) δ 8.21 (s, 1H), 7.41 – 7.14 (m, 5H), 5.24 (bs, 2H), 4.31 (s, 2H), 1.81 (s, 9H). ^{13}C-NMR (75 MHz, DMSO) δ 157.8, 154.7, 154.4, 141.0, 138.2, 129.2, 128.4, 127.3, 100.7, 59.9, 35.2, 29.2. EI-MS (m/z): 225, 281 $[M]^+$. HRMS: calculated mass 282,1713 ; found mass 282,1718 .

*1-tert-butyl-3-(2-chlorobenzyl)-1H-pyrazolo[3,4-d]pyrimidin-4-amine (7**b**)*

Compound **7b** was prepared using general procedure **B** (75%). ^{1}H-NMR (300 MHz, DMSO) δ 8.17 (s, 1H), 7.51 – 7.40 (m, 1H), 7.28 – 7.20 (m, 2H), 7.13 (bs, 2H), 7.07 – 6.99 (m, 1H), 4.43 (s, 2H), 1.64 (s, 9H). 13C-NMR (75 MHz, DMSO) δ 158.2, 154.6, 153.8, 139.2, 136.7, 133.1, 130.1, 129.1, 128.0, 127.1, 100.2, 59.1, 40.3, 40.1, 39.8, 39.45, 39.2, 38.9, 38.67, 31.7, 28.7.EI-MS (m/z): 316 [M]+. HRMS: calculated mass 316,1323 ; found mass 316,1323.

*1-tert-butyl-3-(3-chlorobenzyl)-1H-pyrazolo[3,4-d]pyrimidin-4-amine (7**c**)*

Compound **7c** was prepared using general procedure **B** (52%). ^{1}H NMR (300 MHz, $CDCl_3$) δ 8.28 (s, 1H), 7.28 – 7.24 (m, 2H), 7.24 – 7.17 (m, 1H), 7.12 – 7.05 (m, 1H), 4.88 (bs, 2H), 4.29 (s, 2H), 1.81 (s, 9H). ^{13}C NMR (75 MHz, $CDCl_3$) δ 157.81, 157.41, 154.58, 140.23, 139.83, 135.15, 130.43, 128.49, 127.55, 126.45, 100.62, 60.11, 34.83, 29.17. EI-MS (m/z): 315, 317 $[M]^+$. HRMS: Calculated mass: 316,1326; Found mass: 316,1323.

*3-(3-chlorobenzyl)-1-methyl-1H-pyrazolo[3,4-d]pyrimidin-4-amine (7**d**)*

Compound **7d** was prepared using general procedure **B** (59%).^{1}H-NMR (300 MHz, $CDCl_3$) *δ* 8.29 (s, 1H), 7.37 – 7.09 (m, 4H), 5.42 (bs, 2H), 4.30 (s, 2H), 4.03 (s, 3H). ^{13}C NMR (75 MHz, $CDCl_3$) 157.9, 155.7, 154.1, 142.2, 141.6, 132.9, 130.2, 128.4, 127.3, 126.2, 98.1, 33.1, 32.7. *δ*. EI-MS (m/z): 272, 273 $[M]^+$.

*3-(2-bromobenzyl)-1-tert-butyl-1H-pyrazolo[3,4-d]pyrimidin-4-amine (7**e**)*

Compound **7e** was prepared using general procedure **B** (62%). ^{1}H-NMR (300 MHz, $CDCl_3$) δ 8.28 (s, 1H), 7.67 – 7.59 (m, 1H), 7.24 – 7.08 (m, 2H), 7.04 – 6.96 (m, 1H), 5.06 (bs, 2H), 4.42 (s, 2H), 1.81 (s, 9H). ^{13}C-NMR (75 MHz, $CDCl_3$) *δ* 157.4, 154.5, 139.6, 137.4, 133.0, 130.1, 128.8, 128.1, 124.0, 100.7, 60.1, 34.9, 29.2. EI-MS (m/z): 359, 361 $[M]^+$. HRMS: Calculated mass: 360,0819; Found mass: 360,0815.

*3-(3-bromobenzyl)-1-tert-butyl-1H-pyrazolo[3,4-d]pyrimidin-4-amine (7**f**)*

Compound **7f** was prepared using general procedure **B** (83%).^{1}H-NMR (300 MHz, DMSO) *δ* 8.15 (s, 1H), 7.51 (s, 1H), 7.43 – 7.33 (m, 1H), 7.33 – 7.00 (m, 4H), 4.37 (s, 2H), 1.70 (s, 9H).^{13}C-NMR (75 MHz, DMSO) *δ* 158.1, 154.6, 153.9, 142.1, 140.2, 131.2, 130.5, 129.0, 127.4, 121.6, 99.6, 59.2, 32.7, 28.8..EI-MS (m/z): 360 $[M]^+$. HRMS: calculated mass 360,0819 ; found mass 360,0814 .

*1-tert-butyl-3-(2,3-dichlorobenzyl)-1H-pyrazolo[3,4-d]pyrimidin-4-amine (7**g**)*

Compound **7g** was prepared by using general procedure **B** (55%). ^{1}H-NMR (300 MHz, DMSO) *δ* 8.17 (s, 1H), 7.51 – 7.40 (m, 1H), 7.28 – 7.20 (m, 2H), 7.13 (bs, 2H), 7.07 – 6.99 (m, 1H), 4.43 (s, 2H), 1.64 (s, 9H). ^{13}C-NMR (75 MHz, DMSO) δ 158.2, 154.6, 153.8, 139.2, 136.7, 133.1, 130.1, 129.1, 128.0, 127.1, 100.2, 59.1, 40.3, 40.1, 39.8, 39.45, 39.2, 38.9, 38.67, 31.7, 28.7.EI-MS (m/z): 350, 352 $[M]^+$. HRMS: calculated mass 350,09337 ; found mass 350.0934.

*1-tert-butyl-3-(3,4-dichlorobenzyl)-1H-pyrazolo[3,4-d]pyrimidin-4-amine (7**h**)*

Compound **7h** was prepared using general procedure **B** (57%). ^{1}H-NMR (300 MHz, $CDCl_3$) *δ* 8.27 (s, 1H), 7.48 – 7.20 (m, 2H), 7.11 – 6.98 (m, 1H), 5.22 (bs, 2H), 4.27 (s, 2H), 1.80 (s, 9H). ^{13}C-NMR (75 MHz, $CDCl_3$) *δ* 157.5, 154.7, 154.5, 139.3, 138.6, 133.3, 131.4, 131.0, 130.2, 127.6, 100.6, 60.2, 34.3, 29.2. EI-MS (m/z): 341, 351 $[M]^+$. HRMS: Calculated mass: 350,0934; Found mass: 350,0930.

*1-tert-butyl-3-(3-phenylpropyl)-1H-pyrazolo[3,4-d]pyrimidin-4-amine (7**i**)*

Compound **7i** was prepared using general procedure **B** (70%). ^{1}H NMR (300 MHz, $CDCl_3$) *δ* 8.25 (s, 1H), 7.38 – 7.16 (m, 5H), 5.61 (bs, 1H), 2.88 (t, *J* = 7.0 Hz, 2H), 2.76 (t, *J* = 7.2 Hz, 2H), 2.17 – 2.04 (m, 2H), 1.77 (s, 9H).^{13}C NMR (151 MHz, $CDCl_3$) *δ* 158.1, 154.1, 154.1, 142.2, 141.7, 128.7, 128.5, 126.1, 100.4, 59.8, 35.2, 30.9, 29.2, 28.1. EI-MS (m/z): 309 $[M]^+$. HRMS: Calculated mass: 324,2183; Found mass: 324,2180.

*1-methyl-3-(3-phenylpropyl)-1H-pyrazolo[3,4-d]pyrimidin-4-amine (7**j**)*

Compound **7j** was prepared using general procedure **B** (70%). ^{1}H-NMR (400 MHz, $CDCl_3$) *δ* 8.33 (s, 1H), 7.38 – 7.30 (m, 2H), 7.27 – 7.21 (m, 3H), 5.19 (bs, 2H), 3.98 (s, 3H), 2.91 (t, *J* = 7.1 Hz, 2H), 2.79 (t, *J* = 7.1 Hz, 2H), 2.18 – 2.08 (m, 2H). ^{13}C-NMR (101 MHz, $CDCl_3$) *δ* 157.6, 155.8, 154.5, 144.3, 141.4, 128.7, 128.6, 126.2, 99.1, 35.1, 33.5, 30.9, 28.0. EI-MS (m/z): 267 $[M]^+$. HRMS: calculated mass 268,1557 ; found mass 268,1558.

*1-tert-butyl-3-(4-phenylbutyl)-1H-pyrazolo[3,4-d]pyrimidin-4-amine (**7k**)*

Compound **7k** was prepared using general procedure **B** (77%). ^{1}H NMR (300 MHz, $CDCl_3$) δ 8.28 (s, 1H), 7.33 – 7.23 (m, 2H), 7.22 – 7.12 (m, 3H), 5.32 (bs, 2H), 2.90 (t, J = 7.4 Hz, 2H), 2.69 (t, J = 6.9 Hz, 2H), 1.85 – 1.77 (m, 4H), 1.76 (s, 9H). ^{13}C NMR (75 MHz, $CDCl_3$) δ 157.8, 154.3, 154.2, 142.2, 142.1, 128.4, 128.4, 125.9, 100.4, 59.8, 35.3, 30.6, 29.2, 29.2, 28.5. EI-MS (m/z): 323 $[M]^+$. HRMS: Calculated mass: 310,2026; Found mass: 310,2025.

*1-methyl-3-(4-phenylbutyl)-1H-pyrazolo[3,4-d]pyrimidin-4-amine (**7l**)*

Compound **7l** was prepared using general procedure **B** (52%). ^{1}H-NMR (300 MHz, DMSO) δ 8.14 (s, 1H), 7.39 – 7.02 (m, 5H), 3.80 (s, 3H), 2.95 (s, 2H), 2.60 (s, 2H), 1.65 (s, 4H). ^{13}C-NMR (75 MHz, DMSO) δ 158.0, 155.4, 153.9, 144.4, 142.12, 128.21, 128.17, 125.6, 98.0, 34.89, 33.0, 30.3, 28.4, 27.4. EI-MS (m/z): 281 $[M]^+$. HRMS: Calculated mass: 282,1713; Found mass: 282,1712.

*1-tert-butyl-3-(1-naphthylmethyl)-1H-pyrazolo[3,4-d]pyrimidin-4-amine (**1**)*

Compound **1** was prepared using general procedure **B** (50%). ^{1}H NMR (300 MHz, $CDCl_3$) δ 8.26 (s, 1H), 8.24 – 8.16 (m, 1H), 7.94 – 7.86 (m, 1H), 7.85 – 7.77 (m, 1H), 7.61 – 7.48 (m, 2H), 7.45 – 7.34 (m, 1H), 7.23 – 7.14 (m, 1H), 4.82 (bs, 2H), 4.76 (s, 2H), 1.84 (s, 9H). ^{13}C NMR (75 MHz, $CDCl_3$) δ 157.6, 154.7, 154.5, 140.6, 134.0, 133.9, 131.9, 128.9, 128.2, 126.6, 126.2, (125.8, 125.6, 123.5, 101.1, 60.0, 32.7, 29.2. EI-MS (m/z): 331 $[M]^+$. HRMS: Calculated mass: 332,1870; Found mass: 332,1866.

*1-methyl-3-(1-naphthylmethyl)-1H-pyrazolo[3,4-d]pyrimidin-4-amine (**7m**)*

Compound **7n** was prepared using general procedure **B** (56%). ^{1}H-NMR (300 MHz, $CDCl_3$) δ 8.29 (s, 1H), 8.20 – 8.10 (m, 1H), 7.96 – 7.88 (m, 1H), 7.87 – 7.80 (m, 1H), 7.58 – 7.51 (m, 2H), 7.46 – 7.36 (m, 1H), 7.31 – 7.27 (m, 1H), 4.83 (s, 2H), 4.77 (s, 2H), 4.07 (s, 3H). ^{13}C-NMR (100 MHz, $CDCl_3$) δ 157.5, 155.9, 155.0, 143.0, 134.1, 133.5, 131.9, 129.0, 128.5, 126.8, 126.3, 126.2, 125.6, 123.5, 99.7, 33.7, 32.8. EI-MS (m/z): 289 $[M]^+$. HRMS: Calculated mass: 290,1400; Found mass: 290,1403.

*2,4,6-trichloropyrimidine-5-carbaldehyde (**8**)*

$POCl_3$ (58.2mL, 0.62mol) was added dropwise over 20 min. to a flask cooled in an ice bath and charged with DMF (10 mL). The solution was stirred for 30 min. after which barbituric acid (10g, 0.078 mmol) was added in one portion. The solution was left to stir at room temperature for 1 hr. Next the reaction mixture was heated to 100°C for 4 hrs. The reaction mixture was evaporated under reduced pressure to half-volume, after which it was poured over ice while still warm. Water was added and the suspension was stirred for 10 min. The yellow precipitate was filtered off and washed with water. The residue was triturated with hexane. After filtration the white crystals were dried to obtain 11.05g of the title compound (67%) ^{1}H-NMR (300 MHz, $CDCl_3$) δ 10.42 (s, 1H). ^{13}C-NMR (75 MHz, $CDCl_3$) δ 184.6, 164.1, 161.6, 123.0. CI-MS (m/z): 245, 247 $[M+H]^+$.

*1-tert-butyl-4,6-dichloro-1H-pyrazolo[3,4-d]pyrimidine (**9**)*

To a solution of 2,4,6-trichloropyrimidine-5-carbaldehyde (**8**) (0.5g, 2.365 mmol) in 10mL MeOH, was added dropwise a solution of *tert*-butylhydrazine hydrochloride (0.324g, 2.6 mmol) in 5mL MeOH and a solution of Et_3N (0.359ml, 4.73 mmol) in 5mL MeOH subsequently at 0°C. Afterwards the reaction mixture was left to stir for 3 hrs. The solvent was evaporated *in vacuo* after which the residue was purified using MPLC (9/1

Hept/EtOAc v/v) to yield 515mg of the title compound as a pale white solid (89%). ^{1}H-NMR (300 MHz, $CDCl_3$) δ 8.08 (s, 1H), 1.81 (s, 9H). ^{13}C-NMR (75 MHz, $CDCl_3$) δ 155.1, 155.0, 153.7, 130.8, 113.8, 62.0, 29.1. CI-MS (m/z): 245, 247 $[M+H]^+$. HRMS: Calculated mass: 245,0355; Found mass: 245,0353.

*1-tert-butyl-6-chloro-1H-pyrazolo[3,4-d]pyrimidin-4-amine (**10**)*

To a solution of 1-*tert*-butyl-4,6-dichloro-1*H*-pyrazolo[3,4-*d*]pyrimidine (**10**) (1g, 4.08mmol) in dioxane (40mL), was added a 25% weight solution of NH_3 in H_2O (89 mL). The reaction mixture was stirred for 3 hrs at reflux. Next the solvent was evaporated *in vacuo* to obtain 0.87g of the title compound as a white solid (95%). ^{1}H-NMR (300 MHz, $CDCl_3$) δ 7.83 (s, 1H), 5.67 (bs, 2H), 1.77 (s, 9H). ^{13}C-NMR (75 MHz, $CDCl_3$) δ 158.7, 156.0, 153.5, 130.6, 100.3, 59.6, 28.7. EI-MS (m/z): 225 $[M]^+$.

*N^6-butyl-1-tert-butyl-1H-pyrazolo[3,4-d]pyrimidine-4,6-diamine (**11**)*

A pressure tube was charged with 1-*tert*-butyl-6-chloro-1*H*-pyrazlo[3,4-*d*]pyrimidin-4-amine (**11**) (0.2g, 0.886 mmol) and *n*-butylamine (3 mL, 31 mmol). The reaction mixture was heated at 80°C for 12 hrs. After TLC showed full conversion, the reaction was quenched with an aqueous solution of $NaHCO_3$ and extracted with DCM (3x). Next the organic layers were combined and dried over $MgSO_4$, filtered. The solvents were removed in vacuo after which the residue was purified using MPLC (7/4 Hept/EtOAc v/v) to yield 185mg of the title compound as a white solid. (80%) ^{1}H-NMR (300 MHz, $CDCl_3$) δ 7.63 (s, 1H), 5.15 (bs, 2H), 4.92 (bs, 1H), 3.43 (q, J = 12.9, 7.3 Hz, 2H), 1.68 – 1.53 (m, 2H), 1.50 – 1.35 (m, 2H), 0.96 (t, J = 7.3 Hz, 3H). ^{13}C-NMR (75 MHz, $CDCl_3$) δ 160.2, 157.5, 155.9, 128.9, 97.0, 59.2, 41.3, 31.8, 28.8, 20.2, 13.9. EI-MS (m/z): 262 $[M]^+$. HRMS: Calculated mass: 263,1979; Found mass: 263,1976.

*3-bromo-N^6-butyl-1-tert-butyl-1H-pyrazolo[3,4-d]pyrimidine-4,6-diamine (**12**)*

To a soluion of N^6-butyl-1-*tert*-butyl-1*H*-pyrazolo[3,4-*d*]pyrimidine-4,6-diamine (**12**) (0.2g, 0.762 mmol) in DCM (8 mL) was added Br_2 (0.157ml, 3.05 mmol) while cooling in an ice bath. The reaction mixture was stirred for 1 hr at room temperature afer which it was refluxed for 3hrs. TLC analysis showed full conversion. The reaction was quenched with a 10% aqueous solution of $Na_2S_2O_3$-solution and extracted with DCM (3x). Next the organic layers were dried with $MgSO_4$, filtered and the solvent was removed *in vacuo*. The residue was purified using MPLC (Hept/EtOAc 7/3 v/v) to yield 200 mg of the title compound as a yellow solid. (77%) ^{1}H-NMR (300 MHz, $CDCl_3$) δ 5.46 (s, 2H), 4.95 (s, 1H), 3.68 – 3.20 (m, 2H), 1.72 (s, 9H), 1.67 – 1.54 (m, 2H), 1.50 – 1.35 (m, 2H), 0.96 (t, J = 7.3 Hz, 3H). ^{13}C-NMR (75 MHz, $CDCl_3$) δ 160.6, 157.2, 156.56, 115.7, 96.4, 60.2, 41.2, 31.7, 28.9, 20.2, 13.9. EI-MS (m/z): 341, 344 $[M]^+$.

*N^6-butyl-1-tert-butyl-3-phenyl-1H-pyrazolo[3,4-d]pyrimidine-4,6-diamine (**13a**)*

Compound **13a** was prepared using general procedure **A** to obtain 26 mg of a yellow oil (52%). The oil was crystallized via the generation of the HCl salt via addition of a Et_2O-solution saturated with HCl. After filtration a white solid was obtained. ^{1}H-NMR (300 MHz, $CDCl_3$) δ 7.75 – 7.63 (m, 2H), 7.55 – 7.37 (m, 3H), 5.08 (bs, 2H), 4.97 (bs, 1H), 3.46 (q, J = 12.9, 7.0 Hz, 2H), 1.81 (s, 9H), 1.71 – 1.55 (m, 2H), 1.54 – 1.36 (m, 2H), 0.98 (t, J = 7.3 Hz, 3H). ^{13}C-NMR (75 MHz, $CDCl_3$) δ 160.0, 157.8, 156.9, 142.4, 134.5, 129.1, 128.5, 128.4, 94.9, 59.3, 41.3, 31.8, 28.9, 20.2, 13.9. EI-MS (m/z): 338 $[M]^+$. HRMS: Calculated mass: 339,2292; Found mass: 339,2288

*N^6-butyl-1-tert-butyl-3-(3-chlorophenyl)-1H-pyrazolo[3,4-d]pyrimidine-4,6-diamine (**13b**)*

Compound **13b** was prepared using general procedure **A** to obtain 40 mg of a yellow oil (73%). The oil was crystallized via the generation of the HCl salt via addition of a Et_2O-solution saturated with HCl. After filtration a white solid was obtained. ^{1}H-NMR (300 MHz, $CDCl_3$) δ 7.73 – 7.68 (m, J = 1.9 Hz, 1H), 7.60 – 7.52 (m, 1H), 7.47 – 7.35 (m, 2H), 5.06 (bs, 2H), 4.92 (bs, 1H), 3.45 (q, J = 12.9, 7.0 Hz, 2H), 1.80 (s, 9H), 1.70 – 1.56 (m, 2H), 1.52 – 1.37 (m, 2H), 0.98 (t, J = 7.3 Hz, 3H). ^{13}C-NMR (75 MHz, $CDCl_3$) δ 160.2, 157.7, 157.1, 140.8, 136.3, 135.0, 130.2, 128.6, 128.4, 126.5, 94.8, 59.5, 41.2, 31.8, 28.9, 20.2, 13.9. EI-MS (m/z): 371, 373 $[M]^+$. HRMS: Calculated mass: 373,19018; Found mass: 373,1897.

*N^6-butyl-1-tert-butyl-3-(4-fluorophenyl)-1H-pyrazolo[3,4-d]pyrimidine-4,6-diamine (**13c**)*

Compound **13c** was prepared using general procedure **A** to obtain 31 mg of a yellow solid (59%). ^{1}H-NMR (300 MHz, DMSO) δ 7.70 – 7.55 (m, 2H), 7.43 – 7.24 (m, 2H), 6.57 (bs, 1H), 6.08 (bs, 2H), 3.28 (q, J = 13.2, 6.8 Hz, 2H), 1.69 (s, 9H), 1.63 – 1.45 (m, 2H), 1.41 – 1.27 (m, 2H), 0.91 (t, J = 7.3 Hz, 3H). ^{13}C-NMR (75 MHz, DMSO) δ 162.5 (d, ^1J = 244.6 Hz), 160.8(s) , 158.5(s), 157.4(s), 141.1(s), 131.0 (d, ^4J = 3.0 Hz), 130.6 (d, ^3J = 8.3 Hz), 116.2 (d, ^2J = 21.6 Hz), 94.4(s), 58.9(s), 55.4(s), 31.8(s), 29.0(s), 20.2(s), 14.3(s). EI-MS (m/z):299, 356 $[M]^+$. HRMS: calculated mass 357,2197 ; found mass 357,2194.

*N^6-butyl-1-tert-butyl-3-(4-methoxyphenyl)-1H-pyrazolo[3,4-d]pyrimidine-4,6-diamine (**13d**)*

Compound **13d** was prepared using general procedure **A** to obtain 28 mg of a yellow oil (52%). ^{1}H NMR (300 MHz, $CDCl_3$) δ 7.65 – 7.56 (m, 2H), 7.07 – 6.98 (m, 2H), 5.21 (bs, 2H), 4.96 (bs, 1H), 3.87 (s, 3H), 3.45 (q, J = 12.9, 7.0 Hz, 2H), 1.79 (s, 9H), 1.70 – 1.56 (m, 2H), 1.51 – 1.37 (m, 2H), 0.97 (t, J = 7.3 Hz, 3H). ^{13}C NMR (75 MHz, $CDCl_3$) δ 159.9, 159.8, 157.8, 156.7, 142.2, 129.7, 126.8, 114.5, 94.8, 59.3, 55.34, 41.2, 31.8, 28.9, 20.12, 13.9. EI-MS (m/z): 368 $[M]^+$. HRMS: Calculated mass: 369,2397; Found mass: 369,2392.

*N^6-butyl-1-tert-butyl-3-(3-thienyl)-1H-pyrazolo[3,4-d]pyrimidine-4,6-diamine (**13e**)*

Compound **13e** was prepared using general procedure **A** to obtain 39 mg of a yellow oil (77%). The oil was crystallized via the generation of the HCl salt via addition of a Et_2O-solution saturated with HCl. After filtration a white solid was obtained. ^{1}H NMR (300 MHz, $CDCl_3$) δ 7.60 – 7.52 (m, 1H), 7.51 – 7.38 (m, 2H), 5.17 (s, 2H), 4.92 (s, 1H), 3.46 (q, J = 13.2, 6 Hz, 2H), 1.71 – 1.53 (m, 2H), 1.52 – 1.33 (m, 2H), 0.97 (t, J = 7.3 Hz, 3H). ^{13}C NMR (75 MHz, $CDCl_3$) δ 160.0, 157.8, 156.7, 137., 135.4, 127.9, 127.0, 123.5, 95.1, 59.3, 41.2, 31.8, 28.9, 20.12, 13.9. EI-MS (m/z): 344 $[M]^+$. Calculated mass: 345,1856; Found mass: 345,1855.

*N^6-butyl-1-tert-butyl-3-[3-(trifluoromethyl)phenyl]-1H-pyrazolo[3,4-d]pyrimidine-4,6-diamine (**13f**)*

Compound **13f** was prepared using general procedure **A** to obtain 26 mg of a yellow oil (52%). ^{1}H-NMR (300 MHz, $CDCl_3$) δ 7.98 (s, 1H), 7.92 – 7.85 (m, 1H), 7.73 – 7.56 (m, 2H), 4.98 (bs, 2H), 4.91 (s, 1H), 3.55 – 3.36 (q, J = 12.9, 7.0 Hz, 2H), 1.81 (s, 9H), 1.70 – 1.58 (m, 2H), 1.54 – 1.36 (m, 2H), 0.98 (t, J = 7.3 Hz, 3H). ^{13}C-NMR (150 MHz, $CDCl_3$) δ 160.1 (s), 157.6 (s), 157.2 (s), 140.8 (s), 135.3 (q, ^4J = 3.2 Hz), 131.6 (q, ^4J = 2.6 Hz), 131.3 (q, ^2J = 21.2 Hz), 129.5 (s), 125.4 (q, ^3J = 7.6 Hz), 125.0 (q, ^3J = 7.6 Hz), 122.1 (q, ^1J = 246.0 Hz), 94.9 (s), 59.6 (s), 41.3 (s), 31.8 (s), 28.9 (s), 20.2 (s), 13.9 (s). EI-MS (m/z): 407 $[M]^+$. Calculated mass: 407,2165; Found mass: 407,2161.

*N^6-butyl-1-tert-butyl-3-(2-naphthyl)-1H-pyrazolo[3,4-d]pyrimidine-4,6-diamine (**13g**)*

Compound **13g** was prepared using general procedure **A** starting from 38 mg of the starting product to obtain 31 mg of a yellow oil (71.7%). ^{1}H NMR (300 MHz, DMSO) δ 8.13 (s, 1H), 8.08 – 7.92 (m, 3H), 7.82 – 7.72 (m, 1H), 7.61 – 7.51 (m, 2H), 6.61 (bs, 1H), 6.14 (bs, 2H), 3.34 – 3.23 (m, J = 13.5, 7.0 Hz, 2H), 1.73 (s, 9H), 1.63 – 1.50 (m, 2H), 1.42 – 1.29 (m, 2H), 0.91 (t, J = 7.3 Hz, 3H). ^{13}C NMR (75 MHz, DMSO) δ 160.3, 158.1, 156.8, 141.5, 133.0, 132.5, 131.6, 128.4, 128.1, 127.6, 126.9, 126.5, 126.3, 126.2, 94.1, 58.5, 41.5, 31.3, 28.5, 19.7, 13.8. EI-MS (m/z): 388 $[M]^+$.

*N^6-butyl-1-tert-butyl-3-(1-naphthyl)-1H-pyrazolo[3,4-d]pyrimidine-4,6-diamine (**13h**)*

Compound **13h** was prepared by using general procedure **A** (65%). ^{1}H-NMR (300 MHz, DMSO) δ 8.10 – 8.01 (m, 2H), 7.98 – 7.91 (m, 1H), 7.70 – 7.49 (m, 4H), 6.61 (s, 1H), 3.35 – 3.27 (m, 2H), 1.76 (s, 9H), 1.64 – 1.52 (m, 2H), 1.44 – 1.30 (m, 2H), 0.94 (d, J = 7.3 Hz, 3H). ^{13}C-NMR (75 MHz, DMSO) δ 160.5, 157.7, 156.2, 139.8, 133.5, 131.4, 131.2, 128.7, 128.3, 127.8, 126.6, 126.2, 125.7, 125.5, 95.8, 58.5, 31.3, 28.6, 19.7, 13.8. EI-MS (m/z): 388, 331 $[M]^+$. HRMS: calculated mass 389,2448; found mass 389,2444.

*3-bromo-1H-indole (**14**)*

A 25 mL RB-flask was charged with indole (0.5g, 4.27 mmol) in 5 mL pyridine. Next pyridinium tribromide (1.7g, 5.34 mmol) in 5 mL pyridine was added dropwise over 10 minutes after which the reaction was left to stir overnight at rt. Next ice water was added and the reaction mixture was extracted with Et_2O (3x). The OL was washed with 6N HCl and saturated $NaHCO_3$, dried with $MgSO_4$, filtered and concentrated in vacuo to be used as such.

*3-bromo-1-(phenylsulfonyl)-1H-indole (**15**)*

A 2-necked flask was charged with 3-bromo-1H-indole (**14**) (400mg, 2,04mmol) and TBAHS (104mg, 0.306mmol) in 20mL toluene at 0°C. Next a 50% aqueous NaOH-solution (20mL) and benzenesulfonyl chloride (541mg, 3.06 mmol) were added dropwise. The reaction mixture was left to stir at rt overnight. After TLC showed full conversion, the OL layer was separated, acidified with 1N HCl, neutralized with NaHCO3, washed with brine, dried with MgSO4. The crude product was purified using MPLC (heptane/EtOAc, 9/1, v/v) to obtain 612mg of the desired product (89%).^{1}H-NMR (300 MHz, DMSO) δ 8.16 (s, 1H), 8.08 – 7.98 (m, 3H), 7.76–7.68 (m, 1H), 7.67 – 7.57 (m, 2H), 7.52 – 7.34 (m, 3H). ^{13}C-NMR (75 MHz, DMSO) δ 136.5, 134.9, 133.5, 130.0, 129.0, 126.8, 126.1, 125.6, 124.4, 119.7, 113.4, 98.9. EI-MS (m/z): 337 $[M]^+$

*1-(phenylsulfonyl)-3-(4,4,5,5-tetramethyl-1,3,2-dioxaborolan-2-yl)-1H-indole (**16**)*

A 10 mL RB flask was charged with 3-bromo-1-(phenylsulfonyl)-1H-indole (**15**) (500mg, 1.487mmol), bis(pinacolato)diboron (453mg, 1.785mmol) and KOAc (438mg, 4.46mmol) in 5mL DMF. The reaction mixture was degassed for 20 min, after which $PdCl_2$(dppf) (36,4mg, 3mol%) was added. The reaction was stirred at 80°C overnight. After TLC showed full conversion, EtOAc was added and the mixture was filtered over a path of celite. The OL was evaporated in vacuo. The crude product was purified using MPLC (heptane/EtOAc, 8/2, v/v) to obtain 335mg of the desired product (59%).^{1}H-NMR (300 MHz, DMSO) δ 8.09 (d, J = 7.6 Hz, 1H), 8.00 – 7.90 (m, 1H), 7.82 (d, J = 7.6 Hz, 1H), 7.72 (t, J = 7.2 Hz, 1H), 7.62 (t, J = 7.7 Hz, 1H), 7.41 – 7.25 (m, 1H),

1.32 (s, 6H). ^{13}C-NMR (75 MHz, DMSO) δ 136.9, 134.7, 133.8, 132.8, 123.0, 127.89, 127.0, 124.8, 123.7, 122.6, 112.9, 83.5, 24.6. EI-MS (m/z): 383 $[M]^+$

*1-tert-butyl-3-[1-(phenylsulfonyl)-1H-indol-3-yl]-1H-pyrazolo[3,4-d]pyrimidin-4-amine (**17**)*

A 10 mL flask was charged with 3.4 (50mg, 0.185 mmol), 1-(phenylsulfonyl)-3-(4,4,5,5-tetramethyl-1,3,2-dioxaborolan-2-yl)-1*H*-indole (**16**) (78mg, 0.204 mmol) and Na_2CO_3 (39.2mg, 0.370 mmol) in dioxane/H_2O (1mL/0.25mL). The vessel was degassed using a Schlenk line(3x). Next $Pd(PPh_3)_2Cl_2$ (5mol%) was added and the reaction mixture was stirred at reflux overnight. The crude product was filtered over a path of celite and rinsed with EtOAc. The filtrate was extracted with H_2O. The combined organic layers were washed with brine, dried with $MgSO_4$, filtered and concentrated in vacuo. The crude product was purified using MPLC (heptane/EtOAc, 7/3, v/v) to obtain 61mg of the desired product (74%). ^{1}H-NMR (300 MHz, DMSO) δ 8.28 (s, 1H), 8.15 – 7.98 (m, 5H), 7.71 (t, J = 7.2 Hz, 1H), 7.60 (t, J = 7.6 Hz, 2H), 7.49 – 7.31 (m, 2H), 7.04 (bs, 2H), 1.77 (s, 9H). ^{13}C-NMR (75 MHz, DMSO) δ 158.6, 154.7, 153.5, 137.0, 134.7, 134.4, 133.9, 129.9, 129.0, 127.0, 125.9, 125.3, 123.9, 121.8, 115.2, 113.1, 99.5, 59.8, 28.7. EI-MS (m/z): 390, 446 $[M]^+$

*1-tert-butyl-3-(1H-indol-3-yl)-1H-pyrazolo[3,4-d]pyrimidin-4-amine (**18**)*

A 5 mL RB flask was charged with 1-*tert*-butyl-3-[1-(phenylsulfonyl)-1*H*-indol-3-yl]-1*H*-pyrazolo[3,4-*d*]pyrimidin-4-amine (3.49) (50mg, 0.112mmol) and TBAF-solution in THF (1M, 0.224ml, 0.224mmol) in 2mL THF. The reaction was stirred overnight at reflux. After TLC showed full conversion, the solvent was evaporated. The crude product was purified using MPLC (heptane/EtOAc, 7/3, v/v) to obtain 25mg of the desired product (73%). ^{1}H-NMR (300 MHz, DMSO) δ 8.70 (bs, 1H), 8.36 (s, 1H), 7.81 (d, J = 7.8 Hz, 1H), 7.52 (d, J = 2.5 Hz, 1H), 7.45 (d, J = 7.8 Hz, 1H), 7.33 – 7.17 (m, 2H), 5.47 (bs, 2H), 1.87 (s, 9H). ^{13}C-NMR (75 MHz, DMSO) δ 158.0, 154.5, 154.0, 136.4, 136.3, 126.3, 123.9, 123.3, 121.0, 120.4, 111.5, 109.8, 100.9, 6.31, 29.3. EI-MS (m/z): 249, 306 $[M]^+$

Production and purification of GST-PKD1/2 in HEK293T cells

HEK293T cells were cultured in DMEM (Sigma Aldrich, St. Louis, USA) supplemented with 1% Glutamax (Gibco, Life Technologies, California, USA), 100U/ml penicillin and 100µg/ml streptomycin (Sigma Aldrich, St. Louis, USA) and 10% Fetal bovine serum (Hyclone, GE Healthcare, Wisconsin, USA). Cells were plated at a density of 2x106 cells plate and transiently transfected with 4µg pDEST27-PRKD1/2 (including stop codon) using polyethylenimine (PEI) (Polysciences Inc., Washington, USA) in a 5:1 (w/w) ratio. 48h after transfection cells were stimulated with 500nM Phorbol 12,13-dibutyrate (PDB) (Sigma Aldrich, St. Louis, USA) for 15 minutes and subsequently lysed in a buffer containing 50mM Tris pH 7.4, 150mM NaCl, 1mM EDTA, 1 mM Na3VO4; 50 mM NaF; 5 mM NaPPi (sodium pyrophosphate); 0,2 µM microcystine; 1 mM AEBSF(4-(2-aminoethyl)-benzenesulfonyl fluoride hydrochloride); 10 µg/ml leupeptin; 15 mM β-mercapto ethanol and 1 % triton X-100 . Insoluble material was removed by centrifugation at 10000g for 15 minutes at 4°C. Next, cell lysate was incubated with gluthation sepharose beads (GE Healthcare, Wisconsin, USA) for 2h at 4°C. The beads were washed 2 times with the aforementioned buffer but with NaCl concentration increased to 500mM. After an extra wash in 50mM Tris, pH7.4, 50mM NaCl, 25% glycerol, proteins were eluted in 50mM Tris pH7.4, 50mM NaCl, 25% glycerol supplemented with 40mM gluthation. Protein concentration was determined

using the Bradford assay using BSA as a standard and purity was determined to be >85% via densitometry scanning of a coomassie stained SDS-polyacrylamide gel.

PKD2 screening assay

An in vitro radiometric assay was used to determine the inhibition of PKD in presence of various compounds at a concentration of 1µM for initial screening, and concentrations ranging from 40µM/20µM to 6nM/3nM for IC50 determinations. Reactions were carried out in 40µl of a mixture containing 50mM Tris-HCl pH 7.4, 10mM $MgCl_2$, 50ng of PDB stimulated GST-PKD2, 4µg/ml Syntide-2 (Sigma Aldrich, St. Louis, USA), 25µM ATP and 2µCi [γ-32P]ATP (Perkin-Elmer, Massachusetts, USA). After 10' the reaction was stopped by spotting 30µl of the reaction on whatmann P81 filter paper. The filter papers were washed 3 times in 0.5% phosphoric acid, followed by one wash in 100% acetone. Subsequently the papers were air-dried and counted using the Tri-Carb 2810 TR scintillation counter (Perkin-Elmer). Percentage of PKD2 inhibition was determined relative to a condition where no inhibitor was added (0,1% dimethylsulfoxide (DMSO)). Graphs were plotted and IC50 values were determined using the GraphPad software package (Prism).

Cellular screening assay using phospho-cortactin as a readout

HEK293T cells were seeded in a 12-well plate at 150.000 cells/well. The next day, cells were transfected with 0.5µg of pcDNA3-Myc-CTTN and 0.5µg of pdcDNA-FLAG-PRKD2. Forty-eight hours after transfection, cells were incubated with compound at the indicated concentrations for 30 min, followed by stimulation with 500nM phorbol 12,13-dibutyrate (PDB) for 15 min. Subsequently, cells were lysed in 50mM Tris pH 7.4, 15mM EDTA, 150mM NaCl, 0.1% (v/v) NP-40 and 25% (v/v) glycerol. Protein quantitation was determined via a Bradford assay. Equal amounts of lysate (30µg) were loaded on a SDS-polyacrylamide gel and subjected to immunoblotting. Blots were probed with Cortactin pSer-298 antibody (home-made) and anti-tubulin antibody (Sigma, St. Louis, Missouri). Subsequently, blots were stripped and reprobed with anti-Myc 9E10 (Sigma, St. Louis, Missouri) for cortactin and anti-FLAG M2 (Sigma, St. Louis, Missouri) for PKD2.

% inhibition of PKD2 at 1µM

Compound	% PKD2 activity remaining (1µM) (average ±SD)	Compound	% PKD2 activity remaining (1µM)
**1**	23 ±1.7	**7f**	59 ±3.3
**6a**	77 ±2.9	**7g**	90 ±5.1
**6b**	81 ±4.0	**7h**	78 ±3.3
**6c**	73 ±2.4	**7i**	101 ±8.3
**6d**	83 ±9.4	**7j**	101±3.3
**6e**	80 ±7.0	**7k**	92.8±5.8
**6f**	81 ±4.5	**7l**	98 ±6.4
**6g**	84 ±9.4	**7m**	64 ±3.9
**6h**	59 ±5.0	**13a**	77 ±5.1
**6i**	34 ±0.4	**13b**	81 ±6.6
**6j**	97 ±0.8	**13c**	69 ±8.3
**6k**	93 ±1.5	**13d**	80 ±2.5
**6l**	53 ±7.1	**13e**	82± 7.2
**7a**	35 ±1.3	**13f**	91 ±8.5
**7b**	41 ±2.6	**13g**	76 ±2.7
**7c**	56 ±2.4	**13h**	89 ±6.2
**7d**	83 ±6.4	**18**	8 ±0.8
**7e**	43 ±6.6		

Enlarged version of figure 5

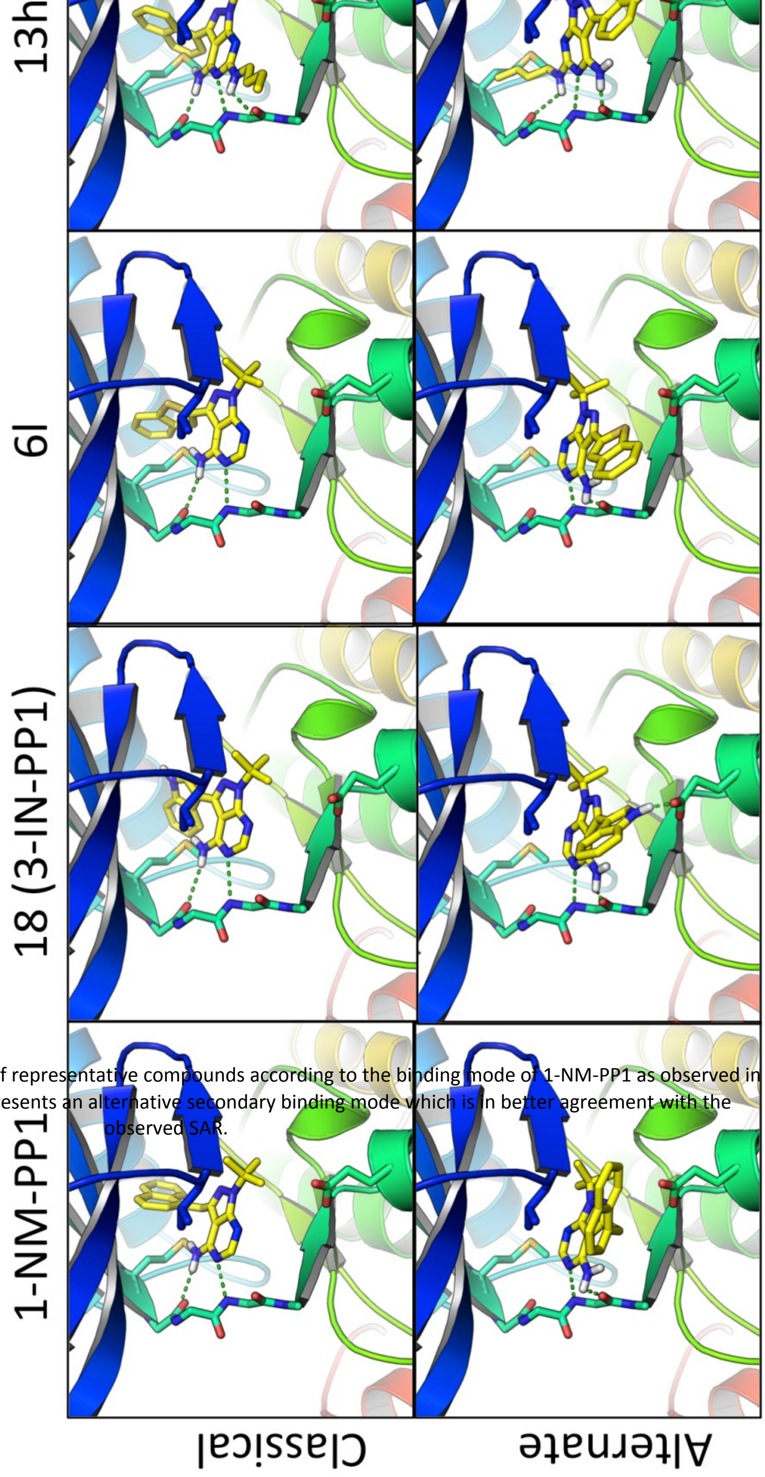

Fig 5 The top row represents the binding mode of representative compounds according to the binding mode of 1-NM-PP1 as observed in classic kinase structures. The bottom row represents an alternative secondary binding mode which is in better agreement with the observed SAR.
